# Application of Next Generation Sequencing for Diagnosis and Clinical Management of Drug-Resistant Tuberculosis: Updates on Recent Developments in the Field

**DOI:** 10.3389/fmicb.2022.775030

**Published:** 2022-03-24

**Authors:** Navisha Dookie, Azraa Khan, Nesri Padayatchi, Kogieleum Naidoo

**Affiliations:** ^1^Centre for the AIDS Programme of Research in South Africa (CAPRISA), University of KwaZulu-Natal, Durban, South Africa; ^2^South African Medical Research Council (SAMRC), CAPRISA HIV-TB Pathogenesis and Treatment Research Unit, Durban, South Africa

**Keywords:** tuberculosis, drug-resistance, next-generation sequencing, whole genome sequencing, targeted sequencing, resistance mechanisms, mixed infection

## Abstract

The World Health Organization’s End TB Strategy prioritizes universal access to an early diagnosis and comprehensive drug susceptibility testing (DST) for all individuals with tuberculosis (TB) as a key component of integrated, patient-centered TB care. Next generation whole genome sequencing (WGS) and its associated technology has demonstrated exceptional potential for reliable and comprehensive resistance prediction for *Mycobacterium tuberculosis* isolates, allowing for accurate clinical decisions. This review presents a descriptive analysis of research describing the potential of WGS to accelerate delivery of individualized care, recent advances in sputum-based WGS technology and the role of targeted sequencing for resistance detection. We provide an update on recent research describing the mechanisms of resistance to new and repurposed drugs and the dynamics of mixed infections and its potential implication on TB diagnosis and treatment. Whilst the studies reviewed here have greatly improved our understanding of recent advances in this arena, it highlights significant challenges that remain. The wide-spread introduction of new drugs in the absence of standardized DST has led to rapid emergence of drug resistance. This review highlights apparent gaps in our knowledge of the mechanisms contributing to resistance for these new drugs and challenges that limit the clinical utility of next generation sequencing techniques. It is recommended that a combination of genotypic and phenotypic techniques is warranted to monitor treatment response, curb emerging resistance and further dissemination of drug resistance.

## Introduction

Globally, tuberculosis (TB) remains a leading cause of mortality from a single infectious agent (WHO, 2020a). The emergence of antimicrobial drug-resistance against *Mycobacterium tuberculosis* (*Mtb*), the causative agent of TB, has exacerbated TB morbidity and mortality worldwide. According to the World Health Organization (WHO) there were 465 000 incident cases of rifampicin resistant-TB (RR-TB) in 2019 (WHO, 2020a). Of these, 78% were multidrug resistant [MDR- defined as resistance to rifampicin (RIF) and isoniazid (INH)] and approximately 2.6% were extensively drug resistant (DR) TB (XDR; defined as MDR-TB isolates with additional resistance to aminoglycosides and fluoroquinolones) (WHO, 2020a). Additionally, one million cases of INH mono-resistant TB were reported for the same period. Despite large-scale global investments for the improvement of laboratory capacity, significant gaps in the detection of resistance were reported in 2019. Only 61% of bacteriologically confirmed TB cases were tested for RR-TB, with only 71% of the notified MDR/RR-TB patients being tested for second-line drug resistance (WHO, 2020a). Without substantial efforts aimed at addressing these diagnostic gaps, global progress toward the End TB strategy goals of an 80% reduction in TB incidence and 90% reduction in global TB deaths by 2030 will not be achieved (WHO, 2020a).

**FIGURE 1:**
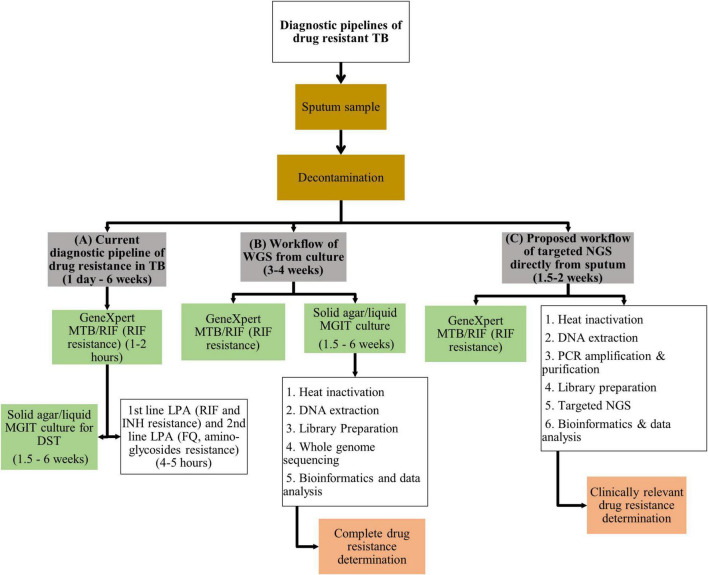
Illustration of **(A)** the current diagnostic pipeline of drug resistance in TB, **(B)** workflow of WGS from culture, and **(C)** proposed workflow of targeted NGS directly from sputum. TB, tuberculosis; WGS, whole genome sequencing; MTB, *Mycobacterium tuberculosis*; MGIT, mycobacterial growth indicator tube; DST, drug susceptibility testing; LPA, line probe assay; RIF, rifampicin; INH, isoniazid; FQ, fluoroquinolones; DNA, deoxyribonucleic acid; NGS, next generation sequencing; PCR, polymerase chain reaction.

The recommended programmatic treatment for DR-TB is delivered through standardized treatment regimens, contributing to amplification of drug-resistance (WHO, 2019b). The ongoing use of standardized regimens is driven by the need to eliminate drug-susceptibility testing (DST) from public health programs (Cox et al., 2018). Limitations to standard culture-based DST include access to and availability of specialized infrastructure, associated high laboratory costs and delays in timely delivery of results (Doughty et al., 2014). The introduction of molecular-based DST for TB has revolutionized TB diagnostics, evidenced by the substantial improvement in the detection of RIF resistance from 7% in 2012 to 61% in 2019 (WHO, 2021a). The most substantial gain emanating from molecular-based technology is the GeneXpert MTB/RIF and GeneXpert Ultra assays (Xpert; Cepheid, Sunnyvale, CA, United States) that detect *Mtb* infection and resistance to RIF within 1–2 h, identifying patients with presumptive MDR-TB (WHO, 2013). The implementation of these platforms has demonstrated significant improvement to intermediate outcomes relating to quality of care such as time to diagnosis and effective therapy (Schumacher et al., 2016). Line probe assays (LPAs) such as GenoType MTBDR*plus* and MTBDR*sl* (Hain Lifescience GmbH, Nehren, Germany) provide rapid information within 4–5 h on specific resistance conferring mutations for selected first and second-line drugs, helping refine DR-TB regimen choice (Hain Lifescience GmbH, 2021a,b). Performance of these genotypic assays to guide appropriate selection of DR-TB treatment does however remain limited (Maiga et al., 2012; Hingley-Wilson et al., 2013; Zetola et al., 2014b; Tagliani et al., 2015; Nathavitharana et al., 2017; Makhado et al., 2018; Guglielmetti et al., 2019; Mishra et al., 2020). None of these assays test for resistance to the new/repurposed drugs such as bedaquiline (BDQ), linezolid (LZD), delamanid (DLM), and clofazimine (CFZ). These drugs are core components of category A, B, and C drugs in WHO’s updated hierarchical classification of DR-TB drugs (WHO, 2019b). While Xpert and LPA’s reduce time to results by bypassing culture in the current diagnostic pipeline of DR-TB (Figure 1A), these molecular assays are limited in the number of drugs or gene regions analyzed. Hence a gap exists in that no assay covers resistance to all the current first-line, second-line, new and repurposed drugs simultaneously.

Next generation sequencing (NGS) technology specifically whole genome sequencing (WGS) for TB offers the most comprehensive approach to molecular-based DST (Iketleng et al., 2018). WGS allows interrogation of all mutations that could potentially confer drug-resistance to the infecting organism thereby enabling treatment individualization (Witney et al., 2015; Cox et al., 2018). WGS has now become the standard of care in well-resourced settings (Pankhurst et al., 2016; Walker et al., 2017; Allix-Béguec et al., 2018; Tagliani et al., 2018). The capacity of WGS to track mixed infections, heteroresistance, transmission patterns, outbreaks and potential superspreaders provides additional clinical advantage (Walker et al., 2013, 2014; Guerra-Assunca̧õ et al., 2015a,b; Hatherell et al., 2016; McNerney et al., 2018; Lee et al., 2020b). Recently published data describing the agreement between Xpert, LPAs and WGS compared to phenotypic DST for predicting resistance to 15 drugs, was reported as 49, 63, and 93%, respectively (Heyckendorf et al., 2018). The adoption of WGS in low-income, high TB-burden settings where it is most needed, is however hampered by the requirement for specialized facilities with complex workflows, personnel with high level expertise and data analysis capability (Mahomed et al., 2017). The overall aim of this review is to capture the recent advances in whole genome sequencing technology, provide an overview on the efforts that have been made toward curating phenotypic and genotypic data for clinical and therapeutic decision making and review advances made in sequencing technology directly from sputum samples to expedite testing so that results become available in a clinically relevant time frame. We further conduct an extensive review on the mechanisms of resistance to new and repurposed drugs for their inclusion into genomic assays and examined the utility of sequencing in diagnosing mixed infections in TB. In addition, further refinement of WGS technology to deliver rapid, point-of-care drug susceptibility in a clinically relevant timeframe is required (Satta et al., 2018). Collectively, these topics contribute to our overall understanding of how sequence-based technology can improve patient centered care.

### Advances in Whole Genome Sequencing Technology

More than two decades following the first complete elucidation of the *Mtb* genome in 1998, sequencing technology has rapidly evolved from its confined use as a research tool to a robust clinical diagnostic platform for DR-TB (Cole et al., 1998; McNerney et al., 2017). Early genomes were sequenced and deciphered using tedious techniques that involved cloning and shotgun sequencing followed by assembly and annotation (Cole et al., 1998). Since availability of high-throughput bench top sequencers and rapid analytical software, WGS technology has become fully automated with reduced cost and complexity (Hess et al., 2020). Table 1 provides an overview of available sequencing platforms and their capabilities. Thousands of *Mtb* genome sequences have emanated from these advances, contributing to better understanding of *Mtb* evolution, TB diagnosis, drug-resistance mutations, optimized treatment, surveillance and transmission (Cohen et al., 2015; Hatherell et al., 2016; Shah et al., 2017; Allix-Béguec et al., 2018; Auld et al., 2018; Zignol et al., 2018). A recent study found a 20% added benefit of WGS over the classic genotypic assays in detecting drug resistance outside the putative DR genes (Lam et al., 2021). Centralizing and linking the sequencing laboratory to the clinical setting could efficiently adopt WGS as a routine assay for TB diagnosis (Cabibbe et al., 2018).

**TABLE 1:** Advantages and disadvantages of whole genome sequencing platforms.

Platform	Read-length	Technique	Advantages	Disadvantages
Illumina (MiSeq)	Short (2 bp × 300 bp)	Amplifies fragmented DNA and primers on a chip holding oligonucleotides with reversible dye-terminator bases before capturing the fluorescently labeled terminator nucleotides using a prepared library.	Allows for antibiotic resistance prediction, construct novel TB genomes, SNP and indel analysis. Short read-lengths enable accurate read data and low per-base error rate. Able to generate paired-end reads (Illumina, 2020). SMOR analysis for determination of low-frequency alleles in complex samples using targeted amplicons and deep sequencing for increased sensitivity over standard WGS (Colman et al., 2015). Superior performance over Ion Torrent (PGM) for GC rich (homopolymer) regions (Zhang G. et al., 2015). High throughput and high sequence yield. Low cost per sample at $22.41 (WHO, 2018b). Low input DNA (1 ng) required for library preparation allowing for use directly on clinical specimens (WHO, 2018b). Shorter sample and library preparation (Loman et al., 2012a).	Long run times and large data output per run. High instrument cost. Short read lengths are problematic for repetitive regions like the PE and PPE gene families which are generally excluded (Meehan et al., 2019).
Ion Torrent (PGM)	Short (400–600 bp)	Semi-conductor detects protons/hydrogen ions that give off an electronic signal during the polymerization of DNA. Uses emulsion PCR, a washing step and library preparation.	Allows for antibiotic resistance prediction, construction of novel TB genomes and full length gene analysis for novel mutations (Daum et al., 2012; ION Torrent Next-Generation Sequencing Technology, 2020). Short read-lengths enable accurate read data and low per-base error rate. Longer read-length compared to Illumina. Short run time and low data output per run. Moderate instrument cost (WHO, 2018b).	Low throughput, few samples per run and requires more hands-on time. Reads obtained are single-stranded (WHO, 2018b). High cost per sample at $48.75 per sample (WHO, 2018b). Requires a compressed nitrogen cylinder and water purification system (WHO, 2018b). Requires preparation of amplified sequence libraries through emulsion PCR and enrichment stages off the instrument (Loman et al., 2012a). Associated with homopolymer errors (Loman et al., 2012b).
Oxford Nanopore Technologies (MinION)	Long (100,000–2 million bp)	Single Molecule Real Time (SMRT) detects change in the ion current when DNA strands pass through the biological nanopore. Depends on library preparation, so user can choose the read length.	Allows for antibiotic resistance prediction, construct novel TB genomes, SNP and indel analysis, genomic rearrangement and nucleotide modifications like cytosine methylation. Longest individual read length enables easier assembly even for un-sheared DNA. Short run time. Lowest instrument cost at $1000. Portable and palm-size instrument. Sample preparation and library steps are shorter (WHO, 2018b). Can continue sequencing until sufficient genome coverage is obtained avoiding sequencing failures from low bacterial loads (Votintseva et al., 2017b). Low cost for high throughput ($16.67 per sample for 54 samples) (WHO, 2018b).	High per base error rate (20–35%) (WHO, 2018b). Has lower single raw read accuracy. Moderate data output per run (WHO, 2018b). Not intended for clinical use. Unable to reliably detect variant frequencies below 40%, requires resistant subpopulations to be at least 50% present in a mixed sample for low-frequency allele detection (Tafess et al., 2019). High cost for lower throughput ($42.86 per sample for 22 samples) (WHO, 2018b).
PacBio (RSII)	Long (60,000 bp)	Single Molecule Real Time (SMRT) uses fluorescence detection.	Allows for antibiotic resistance prediction, construct novel TB genomes, SNP, indel and epigenetic analysis (McNerney et al., 2017; Lee et al., 2019). Long read length enables easier assembly of repetitive gene regions (Ley et al., 2019). Short run time. Low data output per run.	High per base error rate and lowest raw read accuracy. High instrument cost ($750,000) and difficult installation. Moderate throughput and limited capacity to multiplex (WHO, 2018b).

*Bp, base pairs; PGM, Personal Genome Machine; TB, tuberculosis; SNP, single nucleotide polymorphism; SMOR, Single Molecule-Overlapping Reads; SMRT, Single Molecule Real Time; NGS, next generation sequencing; GC, Guanine Cytosine; DNA, deoxyribo-nucleic acid; PE, proline-glutamic acid; PPE, proline-proline-glutamic acid; PCR, polymerase chain reaction.*

The current *Mtb* WGS workflow includes sputum decontamination followed by culture usually in a liquid media system, DNA extraction from culture, library preparation and sequencing using short-read sequencing methods (Figure 1B; WHO, 2018b; Meehan et al., 2019). Sequence analysis involves data validation and quality control, followed by mapping to a reference genome for detection of genomic variation (WHO, 2018b). The lack of an internationally accepted standardized workflow has resulted in inconsistent, incomparable genomic data, underscoring the need for consensus international standards for *Mtb* WGS. Whilst short- and long-read sequencers can reconstruct whole genomes of pathogens, further technical validation ensures that host and non-*Mtb* contaminant reads are removed without eliminating true *Mtb* sequences. Repetitive gene regions including the PP/PPE gene families are generally problematic for short read-sequencers due to challenges associated with mapping repetitive reads and are therefore excluded from analysis (Meehan et al., 2019). However, recent results posted on the preprint server bioRxiv demonstrated that over 65% of these excluded regions can be accurately analyzed on Illumina with high precision (though low recall), besides the PE_PGRS and PPE_MPTR subfamilies (Marin et al., 2021).^1^ Although the function of PE/PPE genes remain unknown, they have been considered important in pathogen-host interaction and virulence exclusive to *Mtb* and should therefore be included in sequencing analyses (Qian et al., 2020). Additional advances in detection and elimination of host and non-*Mtb* contaminants has been expedited through development of advanced software to enable filtration (Egli et al., 2018; Walter et al., 2020). With experience, *Mtb* WGS readouts for other significant criteria such as read depth, base quality, filtering of false positive variants, multilocus sequence typing (MLST), k-mer based-grouping for closest genome matches have been standardized and optimized (Egli et al., 2018). Now with a single well-validated workflow with refinement of recommended steps and the addition of external quality assurance, accuracy of sequence data generated has become more reliable. This is essential as stringent control of meta-data is required to guide DR-TB disease surveillance, generalize outbreak predictions, compare epidemiologic resistance pattern trends and monitor treatment response to mutations observed (Meehan et al., 2019). The advent of the fourth industrial revolution in many low-income settings has helped overcome pre-existing challenges through access to cloud-based data storage services including remote access to WGS analytical software and bioinformatics expertise (Satta et al., 2018).

Tools for resistance prediction and strain lineage typing from reference sequences have been developed to support curated DR-TB mutation database development including Mykrobe Predictor TB, CASTB (the comprehensive analysis server for the *Mycobacterium tuberculosis* complex), KvarQ, PhyResSE (Phylo-Resistance Search Engine), TB profiler, and more recently the MTBSeq (Steiner et al., 2014; Bradley et al., 2015; Coll et al., 2015; Feuerriegel et al., 2015; Iwai et al., 2015; Kohl et al., 2018; Hunt et al., 2019; Phelan et al., 2019). These tools have varying sensitivities, specificities, accuracies, drugs and gene regions analyzed, mutation libraries, strain lineage detection, run times, batch uploads and abilities to read mixed infections and heteroresistance (Schleusener et al., 2017). It is noteworthy, that only TB profiler is capable of excluding phylogenetic markers with no link to resistance. Furthermore, this tool undergoes continuous updates based on emerging literature, including updated resistance profiling for new drugs and regimens (Coll et al., 2015). Recently updated TB Profiler, version 3.0.4, is now compatible with WGS data from long-read sequencers, enabling batch processing, detection of heteroresistance and prediction of resistance to cycloserine (CLS) and the new drugs DLM/Pretomanid (PRT) (Phelan et al., 2019). These tools offer a promise of a future comprehensive point-of-care WGS analysis system, desirable for low-income settings (Table 1).

#### Curating Genomic Data for Clinical Relevance

The interpretation of genomic data for clinical decision-making including selection of appropriate treatment regimen has been challenged by the poor correlation between *Mtb* genomic and phenotypic drug susceptibility results. In the absence of horizontal gene transfer mechanisms, *Mtb* develops resistance primarily through the acquisition of mutations in the core genes and their promoter regions (Sreevatsan et al., 1997; Brosch et al., 2002; Supply et al., 2003). Resistance profiling is achieved by comparing *Mtb* mutations detected to published curated lists. Grading by confidence is determined by genotype-phenotype associations conferred by individual resistance mutations (Miotto et al., 2017; Allix-Béguec et al., 2018; Chesov et al., 2019). Genotype-phenotype correlation has been well established for first-line anti-TB drugs such RIF and INH. Correlative difficulties arise where genetic resistance mechanisms are poorly elucidated, such as with new and repurposed drugs, and where no standardized phenotypic and genotypic correlation exists, such as with older drugs like pyrazinamide (PZA) and ethambutol (EMB) (Walker et al., 2015; Yadon et al., 2017; Allix-Béguec et al., 2018; Bouzouita et al., 2018; Heyckendorf et al., 2018; Miotto et al., 2018). Although advancements in characterizing phenotypic-genotypic correlation have occurred, novel or rare mutations detected outside defined/putative hotspot regions continue to present a diagnostic challenge, as in the case of PZA (Whitfield et al., 2015; Köser et al., 2020). Established gene regions and mutations implicated in resistance for newer drugs have not yet been published because resistance mechanisms remain to be confirmed, as discussed in the chapters below.

Efforts to overcome challenges of genotypic-phenotypic correlation, and access to curated clinical, genomic treatment and outcome data has been achieved through establishment of open access databases such as the Relational Sequencing (ReSeq) TB and the Comprehensive Resistance Prediction for Tuberculosis: and International Consortium (CRyPTIC) databases. These databases comprise large collections of *Mtb* isolates from various geographic locations (Starks et al., 2015; Cirillo et al., 2016). Thus far, the consortium has highlighted the role of WGS compared to routine phenotypic testing in predicting susceptibility to first-line drugs amongst 10 209 isolates from 16 countries that collectively represent all major *Mtb* lineages known (Allix-Béguec et al., 2018). Accurate genotypic predictions were made with sensitivities and specificities above 90% for resistance to RIF, INH, PZA and EMB. The WHO is currently evaluating WGS for routine genotypic DST as the new gold standard for *Mtb* drug-resistance surveillance (WHO, 2018b). Different WGS platforms are currently available that have different capabilities, limitations, read-lengths, cost and data output (Table 1). With advances in the drug development pipeline and WHO guidelines, newer agents aim to overcome all known pre-existing mutations (WHO, 2020b). As we garner understanding of mutations especially from newer drugs and their resistance profiles in different geographic settings, there is a need to constantly update these databases to remain relevant. Funding is also necessitated to sustain ReSeq TB and similar databases. More recently, WHO has issued a comprehensive, curated, catalog of mutations in *Mtb* and their association with resistance. A global dataset of 38 215 isolates representing 74 countries were included in the analysis (WHO, 2021a). In this guideline, a robust algorithmic approach is applied for all mutations and phenotypes based on odds ratios for the association of the mutation with resistance and the positive predictive value using strict criteria, including the bounds of the 95% confidence interval. This criteria however, allows for a mutation to be considered as high confidence if present in at least 5 clinical isolates of *Mtb*. In instances where mutations were distributed all along the length of the whole open reading frame of the gene, the statistical significance of resistance was lower. For the new and repurposed drugs BDQ and CFZ, no significant resistance mutations were reported, largely because these variants are still rare i.e., <90%, thus, have been excluded by the algorithm. Further, the stringent phenotypic DST criteria that was applied precluded BDQ DST techniques as these were not WHO endorsed techniques (WHO, 2021a).

### Sequencing Technology Directly From Sputum

Whole genome sequencing represents the most robust tool to guide DR-TB treatment; however, direct sequencing from clinical specimens remains a major limitation of this technology (McNerney et al., 2017). Expectorated sputum is the most reliable specimen for the diagnosis of pulmonary TB (Siddiqi and Rüsch-Gerdes, 2006). Currently, WGS is conducted on culture from a mycobacterial growth indicator tube (MGIT, BACTEC MGIT 960) system (Figure 1B) because MGIT generates large quantities of bacterial culture (Stinson et al., 2014). However, the slow growth rate of *Mtb* leads to long turn around time to a cultured isolate being ready for WGS. Furthermore working with infectious TB is hazardous and requires specialized laboratory facilities and expertise (Stinson et al., 2014; Gilpin et al., 2016). Additional findings that undermine the accuracy of DST include loss in minor variants that are selective for *Mtb* populations when subcultures are used and variation in genetic diversity from parent isolates when single colonies are cultured (Black et al., 2015; Metcalfe et al., 2017; Doyle et al., 2018; Shockey et al., 2019). The type of culture media used can also influence the growth of different strains compromising the ability to detect mixed strain infection (Hanekom et al., 2013).

Direct sequencing from sputum specimens will allow for rapid and comprehensive resistance profiling in a clinically relevant timeline. However, DNA extraction directly from sputum has numerous challenges. Firstly *Mtb* bacillary load in sputum varies from a few bacilli to millions of organisms (McNerney et al., 2017). Limited numbers of bacilli in sputum, such as in smear negative TB, results in low yield of genomic DNA available for sequencing (Hernández et al., 2015; Nguyen et al., 2019). This problem is not uncommon in HIV endemic settings. Secondly, *Mtb* cell wall is lipid-rich consisting of layers of cross-linked peptidoglycan, lipoglycan and mycolic acids encompassed by waxy coat (Abrahams and Besra, 2018). This robust barrier requires disruption to release genomic DNA for quantification and sequencing. Furthermore, the *Mtb* genome comprises 65–80% cytosine/guanine (GC) content that enhances genomic stability, providing an additional barrier to DNA extraction (Cole et al., 1998; Bryant et al., 2013). Published research show optimized laboratory techniques such as use of enzymatic lysing, bead-beating, heat and enrichment of DR genes to successfully overcome these two challenges (Table 2). Thirdly, an additional challenge of sequencing directly from sputum is DNA contamination of sputum samples from human host cells and other bacteria commonly found in sputum (Cui et al., 2012; Chatterjee et al., 2013). This undermines the ability to obtain sufficient quantities of pure *Mtb* DNA for sequencing. The proposed workflow of sequencing directly from sputum is illustrated in Figure 1C. Various decontamination protocols, cell lysis techniques, DNA extraction methods and enrichment kits have been developed and optimized specifically to overcome these challenges (Table 2; Doughty et al., 2014; Brown et al., 2015; Votintseva et al., 2015, 2017a; Doyle et al., 2018). However, no single study has reported on a technique that is both easily implementable and cost-effective for efficient recovery of viable *Mtb* directly from sputum.

**TABLE 2:** Summary of studies exploring DNA extraction techniques directly from sputum samples applying next generation sequencing technology.

Extraction Technique	Extraction Methodology	Performance of extraction technique and sequencing outcome	Sequencing Platform	Reference
Mechanical bead-beating	Sputum samples (*n* = 43) were centrifuged (16,200 × *g*, 30 min) and pelleted; ribolysis with 50 μl of glass beads (425–600 μm) in FastPrep-24 Classic Instrument (45 s, 6.4 m/s). After adding extraction buffer, proteinase K, vortexing and incubating (56°C, 10 min), DNA was extracted on the LIAISON IXT/Arrow (DiaSorin) automated system utilizing magnetic bead chemistry.	74% of sputum samples generated whole genomes at >85% coverage (majority were smear 3+). Required 5 days from sputum.	WGS on Illumina MiSeq and NextSeq	Doyle et al., 2018
Lysis of human/eukaryotic cells	Smear positive sputum samples (*n* = 40) were decontaminated with NAC-PAC *RED* (Alphatec, United States) and heat killed (95°C, 30 min). DNA extraction by saline wash, MolYsis Basic5 (Molzym Germany) eukaryotic cell lysis, bead beating, ethanol precipitation and GlycoBlue Co-Precipitant for MiSeq sequencing. For MiniSeq and ONT MinION sequencing, samples were saline washed, bead beaten, ethanol precipitated and cleaned with AMPure beads (BC, United Kingdom).	83% of sputum samples yielded sufficient DNA and 65% yielded sequencing data for resistance prediction at >3× coverage. Samples sequenced on the MinION gave >95% genome coverage and >5× depth of coverage. 54% of sputum samples had >12× depth and recovered >90% of the *Mtb* genome.	WGS on Illumina MiSeq, MiniSeq and ONT Minion	Votintseva et al., 2017a
	Smear positive sputum samples (*n* = 8) were NALC/NaOH decontaminated before differential osmotic lysis of human cells in water and digestion with RNase-Free DNase Set (Qiagen). Heat treatment (75°C, 10 min), DNA extraction with NucleoSpin Tissue-Kit (Macherey-Nagel, Germany).	20–99% mapped to the human genome (high contamination) and poor average depth of coverage (adc) (0.002X–0.07X). No samples had sufficient sequencing data. Cost < $69.34 per sample.	Shotgun sequencing on Illumina MiSeq	Doughty et al., 2014
Thermolysis	Smear positive sputum samples (*n* = 24) were NALC/NaOH decontaminated, pelleted, resuspended in PBS then in TE before adding 0.1 mm glass beads. Samples were heat killed (80°C, 50 min), freeze/thawed, vortexed and treated with DNeasy blood and tissue DNA extraction kit (Qiagen).	83% of sputum samples had high quality sequencing data (>20× depth, >90% genome covered). Kit costs $350 per sample. Total lab time of 50–96 h.	WGS on Illumina MiSeq	Brown et al., 2015
	Non-decontaminated sputum samples (*n* = 33) were heat killed (95°C, 1 h), ribolysed and purified with LIAISON Ixt (DiaSorin, Italy) or DNeasy blood and tissue kit (Qiagen, Germany).	At least 85% genome coverage at 20× and average depth of coverage (adc) of 60×.	WGS on Illumina NextSeq	Nimmo et al., 2019
Organic/enzymatic-based methods including CTAB	Smear 3+ sputum samples (*n* = 40; stored at −20°C) were NALC/NaOH decontaminated, boiled (85°C, 10 min) in TE buffer and frozen immediately (−20°C, 15 min). Incubation with lysozyme, proteinase K, SDS and 10% CTAB (1% final concentration). DNA extraction by alcohol precipitation and elution in TE buffer. This was compared to the PrimeXtract DNA extraction kit (Longhorn Vaccines and Diagnostics, United States).	The PrimeXtract DNA extraction kit had higher yields of *Mtb* DNA than CTAB method. DNA yield still too low (<2 μg) for SMRT sequencing.	PacBio sequencing	Ndhlovu et al., 2018
Sure SelectXT Target enrichment (AgiIent)	DNA extracted as described in Brown et al. (2015) above. *Mtb* DNA was enriched with Sure SelectXT Target enrichment (AgiIent) using 120-mer biotinylated RNA baits spanning the entire positive strand of H37Rv.	83% of sputum samples had high quality sequencing data (>20× depth, >90% genome covered). Requires 2–3 days and high-cost ($281.52 per sample).	WGS on Illumina MiSeq	Brown et al., 2015
	DNA extracted as described in Doyle et al. (2018) above. Sure SelectXT Target enrichment (AgiIent) was similarly followed as that of Brown et al. (2015) above including pull-down with streptavidin coated beads. Another reduced set of 120-mer biotinylated RNA baits, designed by Agilent, was used to capture only DR genes (35,960 bp) for MDR-TB sputum samples (*n* = 3) with the ‘Fast’ hybridization buffer (1 h, 65°C).	74% of sputum samples generated whole genomes at >85% coverage (majority were smear 3+) and >2000× average depth of coverage (adc). Required 5 days from sputum and 3 days using a reduced bait set. Streptavidin-coated beads are expensive.	WGS on Illumina MiSeq and NextSeq	Doyle et al., 2018
Deeplex^®^-MycTB assay (Geno Screen, Lille, France)	200 μL of spot sputum samples (*n* = 1494) preserved in 96% ethanol, were digested with proteinase K. Semi-automated Maxwell 16 FFPE DNA purification kit with a Maxwell machine (Promega, United States) was used. The Deeplex^®^d-MycTB assay (GenoScreen, France) amplified PCR products were purified with Agencourt AMPure XP magnetic beads (BC, United States) and DNA quantified by Qubit dsDNA BR assay (Life Technologies, United Kingdom).	Average depth of coverage (adc) of 1349×. 80% of Xpert positive samples were *Mtb* positive by Deeplex. 89% of Deeplex samples had a spoligotype, 49% were assigned lineages and 27% had an incomplete drug susceptibility assessment across target regions due to low bacterial load. 4.7% cases of mixed infections and 4.9% cases of NTMs were detected.	Targeted sequencing (18 genes/15 drugs) on Illumina MiSeq	Kayomo et al., 2020
	Smear positive sputum samples (*n* = 104) were liquefied in OMNIgene sputum reagent (DNA Genotek, Canada) before heat killing (95°C, 30 min) and lysis at 60°C with Maxwell method, as similarly performed by Kayomo et al. (2020)	90.8% of MinION reads while 99.5% of MiniSeq reads mapped to H37Rv. The average depth of coverage (adc) was 4,151× on MinION and 4,177× on MiniSeq. Deeplex amplicons are short for optimal processing on MinION long-read sequencer, therefore higher raw error rates (∼9%). Costs $138.68 per sample for both platforms.	Targeted sequencing (18 genes/15 drugs) on ONT MinION (previously sequenced on Illumina MiSeq)	Cabibbe et al., 2020
Next Generation Rapid DST Assay	Sputum samples (*n* = 12) were NALC/NaOH decontaminated and DNA extracted with GenoLyse^®^(Hain Lifescience, Germany) for the Next Generation rapid DST Assay (Translational Genomics Research Institute, AZ, United States).	Sputum samples could be examined at all 6 gene targets to at least 50,000× coverage in 72 h at ∼$30 per sample for reagent costs.	Targeted sequencing on Illumina MiSeq	Colman et al., 2015

*Mtb, Mycobacterium tuberculosis; adc, average depth of coverage; h, hours; mins, minutes; secs, seconds; Bp, base pairs; Kb, kilo base pairs; Ds, double stranded; BR, broad range; HS, high sensitivity; FFPE, formalin-fixed paraffin-embedded; NALC, N-acetyl-L-cysteine; NaOH, Sodium hydroxide; PBS, Phosphate buffer saline; TE, Tris-Ethylenediamine tetra-acetic acid; SDS, sodium dodecyl sulfate; CTAB, Cetyltrimethylammonium bromide; BC, Beckman Coulter; USA, United States of America; UK, United Kingdom; DR, drug resistant; MDR, multidrug resistant; NTM, non-tuberculosis mycobacteria; DST, drug susceptibility testing; DNA, deoxyribo-nucleic acid; RNA, ribonucleic acid; WGS, whole genome sequencing; PCR, polymerase chain reaction; ONT, Oxford Nanopore Technologies; SMRT, Single-Molecule Real Time.*

### Whole Genome Sequencing vs. Targeted Gene Sequencing for Clinical Utility

The *Mtb* genome comprises of 4,4 million base pairs (bp) which code for approximately 4000 genes (Cole et al., 1998). To date >400 variants of genomic alteration have been described in ∼65 genomic regions with a known or putative link to *Mtb* drug-resistance (Lange et al., 2018). Thus, the entire sequence of the organism has limited utility to clinicians whose main interest is to assess appropriateness of a drug in treating a specific DR form of *Mtb*. An alternative approach for obtaining a comprehensive resistance profile is the design of rapid, targeted genomic panels that include only genes known to be involved in drug-resistance (WHO, 2018b). Targeted panels/amplicons interrogate specific regions in the *Mtb* genome thus its’ depth of coverage and specificity for detecting SNPs with high mutational confidence, including mixed strains, is greatly enhanced over conventional WGS (Tagliani et al., 2017; Makhado et al., 2018; Achkar et al., 2019; Colman et al., 2019; Nimmo et al., 2019). An advantage of this technique is that it overcomes challenges associated with adequate DNA quantity and contamination from host and non-TB DNA obtained directly from sputum (Figure 1C; Brown et al., 2015; Votintseva et al., 2017a). Sputum-based targeted amplicon sequencing can provide clinically relevant rapid information at higher sensitivities to guide therapeutic decisions (Cabibbe et al., 2020; Kayomo et al., 2020). A further compelling gain of deep sequencing is the ability to identify variants at low allele frequencies down to approximately 5%, an advantageous threshold for identifying minor variants and subpopulations (Derache et al., 2018). Recent innovations in targeted sequencing applications are outlined in Table 2. Compared to conventional WGS, it is much less data intensive and requires significantly less data storage (Dilliott et al., 2018). Limitations to targeted amplicon sequencing is the prohibitively high costs that make this method unsuitable for high burden low resource settings. An important limitation is the restricted application of targeted amplicon sequencing to only the known resistance markers, while WGS detects all variations, characterized and uncharacterized, from the reference genome. Uncharacterized mutations to existing drugs, or novel mutations to new drugs require further elucidation to determine the role in conferring resistance for this technique to remain valid. Additionally, there may be other factors that may impact on resistance in the absence of mutations such as efflux pumps responsible for expelling drugs out of the bacterial cell (Ghajavand et al., 2019b).

World Health Organization recently endorsed two new amplicon-based targeted sequencing assays for comprehensive DR-TB resistance profiling directly from sputum (WHO, 2018b). The Next Generation – Rapid DST assay (Translational Genomics Research Institute, United States) screens *Mtb* gene regions for mutations associated with resistance to RIF, INH, fluoroquinolones and second-line injectable drugs (Colman et al., 2015, 2016). The Deeplex^®^-MycTB assay (Genoscreen, Lille, France) is a 24-amplicon mix that has capacity to screen for mutations in 18 genes known to be associated with *Mtb* resistance to 15 anti-TB drugs including the new and repurposed drugs BDQ, CFZ and LZD (Tagliani et al., 2017; Genoscreen, 2019). Detection of resistance to these new drugs is particularly important as these agents are now classified by the WHO as essential drugs in the standard short MDR-TB regimens and emerging short-course DR TB treatment regimen (WHO, 2019a,b; Conradie et al., 2020). Deeplex is the most comprehensive targeted panel offering detection of resistance to these significant drugs. The large coverage depth allows for detection of high-confidence mutations, and detection of minor variants from subpopulations (Tagliani et al., 2017). It is also capable of *Mycobacterium tuberculosis* identification using spacer oligonucleotide typing (spoligotyping). A number of studies successfully used Deeplex in drug resistance determinations, many reporting >90% sequence coverage with this assay (Tagliani et al., 2017; Makhado et al., 2018; Achkar et al., 2019). Cabibbe et al. recently demonstrated the successful application of the Deeplex assay using the portable Oxford Nanopore Technology MinION sequencer (Cabibbe et al., 2020). This combination demonstrated full concordance in the detection of clinically relevant resistance markers compared to conventional sequencing platforms and is a promising solution for point-of-care applicability of sequencing technologies. The Deeplex assay has also been used in nationwide DR-TB surveys and in studies assessing region specific mutations that are not detected by conventional assays (Tagliani et al., 2017; Kayomo et al., 2020). Limitations of the Deeplex assay include poor performance in sputum with low DNA quantity, high costs of procurement of each kit, limited number of gene regions that get assessed – i.e., DLM is not currently assessed and Deeplex throughput is 45 samples per kit which may be too many in low TB burden settings and too few in TB endemic settings.

### Resistance Mechanisms to New and Repurposed Drugs

*Mycobacterium tuberculosis* drug resistance is primarily mediated by acquisition of spontaneous mutations in genes coding for drug targets or in enzymes required for drug activation, not by horizontal gene transfer mechanism (Cole et al., 1998; Castro et al., 2020). Mutations occur predominantly as SNPs and insertions or deletions (Indels) of specific nucleotide bases which provide *Mtb* adaptive survival advantage against potent anti-TB drugs (Brimacombe et al., 2007). Other mechanisms of drug resistance include efflux mediated resistance, deficient DNA repair systems and other compensatory mechanisms (Dookie et al., 2018; Ghajavand et al., 2019b). Epistasis and intrinsic cell wall impermeability further contributes to drug resistance (Al-Saeedi and Al-Hajoj, 2017; Seid and Berhane, 2021). These may be a consequence of sub-optimal concentrations of anti-TB drugs from altered host pharmacokinetics (PK) (Liu et al., 2006; Trauner et al., 2017). Over the last few years, the DR-TB treatment landscape has been transformed with the introduction of new (BDQ, DLM/PRT) and repurposed (LZD and CFZ) drugs used in novel, short-course combinations and a revised hierarchical classification of DR-TB drugs where BDQ, LZD and CFZ constitute the majority of group A and B drugs (WHO, 2016, Ghajavand et al., 2019b; Conradie et al., 2020). Following the revised grouping of drugs and downgrading of the second-line injectable agents, the definition of XDR-TB has since been revised to include resistance to a fluoroquinolone and one other group A drug (WHO, 2021b). Given the importance of these new agents, it is imperative to understand the underlying mechanisms contributing to resistance of these drugs. The following section provides an update on mechanisms that have recently emerged describing resistance to the new and repurposed drugs. Mechanisms of resistance to the conventional anti-TB drugs have been previously reviewed (da Silva and Palomino, 2011; Dookie et al., 2018).

#### Bedaquiline

**FIGURE 2:**
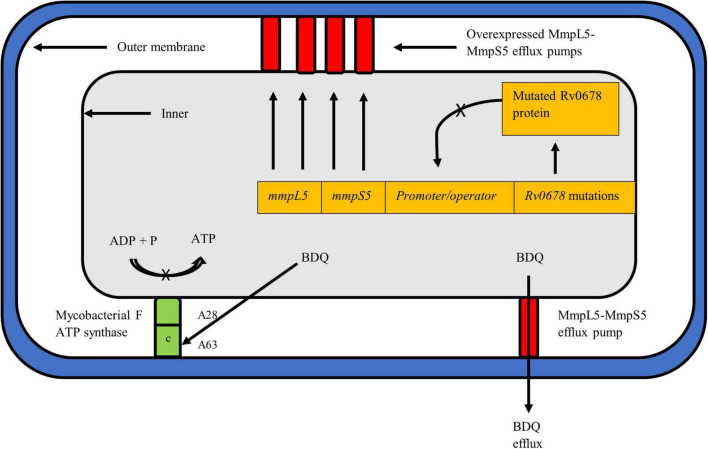
*Mycobacterium tuberculosis* cell depicting the known resistance mechanisms against BDQ, i.e., atpE and Rv0678. The mechanism of action of pepQ, a cytoplasmic peptidase protein, remains to be elucidated. BDQ targets the c subunit of the ATP synth*a*se complex to inhibit ATP production and respiration of mycobacteria. Mutations such as A28 and A63 in atpE prevent BDQ binding to the complex, rendering the drug ineffective. The Rv0678 protein represses mmpS5 and mmpL5 genes to prevent transcription and expression of the MmpS5-MmpL5 efflux pump. A mutated and non-functional Rv0678 repressor cannot bind to the promoter/operator region thereby allowing over-expression of efflux pumps to expel more BDQ out of the mycobacterial cell.

Bedaquiline (Sirturo; previously known as TMC-207 or R207910) is a novel class of anti-TB drugs known as the diaryl-quinolones which inhibits the mycobacterial adenosine triphosphate (ATP) synthase on the *atpE* gene thereby dysregulating pH balance and inhibiting respiration of bacteria (Figure 2; Andries et al., 2005; Koul et al., 2007; Preiss et al., 2015). Preliminary efficacy studies revealed that BDQ significantly reduced the time to culture conversion and demonstrated adequate safety, tolerability and efficacy (Diacon et al., 2012, 2014). The drug, approved by the US-FDA in 2012 for treatment of MDR-TB, has since revolutionized DR-TB treatment forming the core drug of short-course, all-oral treatment for MDR-TB and XDR-TB (Cox and Laessig, 2014; Nunn et al., 2019; Conradie et al., 2020). Clinical trials of DR-TB regimen that contain BDQ administered to MDR- and XDR-TB patients displayed substantially better treatment outcomes, earlier culture conversion and large reduction in TB mortality (Pym et al., 2016; Olayanju et al., 2018; Schnippel et al., 2018; Tweed et al., 2019). Thus given its importance, ongoing surveillance for BDQ resistance needs to be urgently implemented.

Resistance to BDQ has been linked to the mutations occurring in the drug target *atpE*, which encodes the subunit c of the ATP synthase complex (Figure 2; Andries et al., 2005). More recently, mutations in the *atpB* gene, encoding the ATP synthase subunit A, were described in clinical isolates (Martinez et al., 2018). Additional mutations linked to BDQ resistance have been described in the *Rv0678 (mmpR)* and *pepQ (Rv2535c)* off-target genes (Andries et al., 2014; Almeida et al., 2016). Thus far, resistance to BDQ has been studied using a combination of phenotypic susceptibility testing and sequencing techniques to detect genomic alteration. However, given that BDQ is a new drug, the phenotypic criteria for defining BDQ resistance has not been fully characterized (Köser et al., 2019). WHO recommended an interim critical concentration (CC) of 0.25 mg/L for the agar proportion method (Middlebrook 7H11 agar) and 1.0 mg/L when using the MGIT system (WHO, 2018a). A recent external quality assessment conducted in five laboratories across geographically diverse settings helped define the current WHO interim CC value deemed appropriate for BDQ resistance determination (Kaniga et al., 2020). While the WHO suggests use of CC values of 1 mg/L in MGIT and 0.25 mg/L in 7H11, this may be further adjusted and confirmed with emerging data on BDQ (WHO, 2018a).

Using laboratory generated mutants, Andries et al. (2005) first reported two BDQ resistance conferring mutations, A63P and I66M, in the *atpE* gene which resulted in 10–128 fold increase in BDQ MIC. In an analysis of 53 *in vitro* selected mutants, Huitric et al. (2010) subsequently reported three additional mutations (A28V, A28P, and G61A), and also reported that *atpE* gene mutations accounted for only 28% of resistance, indicating presence of additional BDQ resistance mechanisms. Using clinical isolates from patients treated with BDQ, Zimenkov et al. (2017) first reported *atpE* mutations (D28N and A63V) despite these isolates falling within the susceptible MIC range. Recent reports demonstrate A63P/V mutations associated with 4- and 8-fold increase in BDQ MICs among DR-TB isolates (Andres et al., 2020; Peretokina et al., 2020). The E61D mutation also recently emerged for the first time in an MDR-TB patient on BDQ at a 28% variant frequency (BDQ MIC > 2 mg/L in MGIT) (Chesov et al., 2021). In the case of the *atpE* gene, hotspot regions have been identified at codons 28 and 63. Since then a well-characterized catalog of mutations based on an extensive literature review of mutations linked to BDQ resistance has been published. Collectively this data now indicates that *atpE* mutations are rarely identified in clinical isolates (Ismail et al., 2018b). Interestingly, 3 mutations were detected in the *atpB* gene from 1 susceptible and 2 MDR-TB clinical isolates (Martinez et al., 2018). The F222L, H250P and E244K mutations were associated with MICs of 0.031, 0.125, and 8.0 mg/L, respectively, with the latter conferring high-level BDQ resistance.

Non-target based BDQ mutations, notably within the *Rv0678* gene, have since been described as the most common mechanism linked to BDQ resistance (Andries et al., 2014). Rv0678 is a transcriptional mycobacterial membrane protein which represses genes encoding the MmpS5-MmpL5 efflux pump, key to transport of BDQ and CFZ (Figure 2; Andries et al., 2014). Mutations in *Rv0678* inactivate the repressor genes thereby increasing the efflux of both drugs. The MmpS5-MmpL5 efflux pump mechanism is a multisubstrate extrusion mechanism by nature and thus mutations in *Rv0678* results in cross-resistance to CFZ (Hartkoorn et al., 2014). An additional gene, *pepQ*, has also been identified as conferring cross resistance to BDQ and CFZ (Almeida et al., 2016). Detailed description of *pepQ* activity can be found below. The cross resistance in the *Rv0678* and *pepQ* genes between BDQ and CFZ has major implications for the combined use of BDQ and CFZ as recommended in the current WHO short MDR-TB regimen. This is also discussed further in the CFZ section below. In contrast to *atpE* gene mutations, a higher diversity and frequency of mutations have been reported to occur along the entire length of the *Rv0678* gene (Battaglia et al., 2020). Authors postulate that the preferred selection of *Rv0678* over the *atpE* mutation is due to organism ability to retain fitness and overcome the effect of BDQ, despite presence of the *Rv0678* mutation (Andries et al., 2014).

*Rv0678* mutations are dynamic and have been reported at varing frequencies over the course of treatment. Studies have reported low variant frequencies (mutations present at <10%) of acquired BDQ resistance during early treatment, followed by higher variant frequencies during treatment, the latter correlating with an increased BDQ MIC (Trauner et al., 2017; De Vos et al., 2019; Ghodousi et al., 2019; Polsfuss et al., 2019; Nimmo et al., 2020b). However, *Rv0678* variants at low to intermediate frequencies have a variable effect on BDQ MICs challenging clinically relevant breakpoints and cut-off values. Vargas et al. (2021) inferred that only variants present at a frequency of 19% or more will likely become dominant within bacterial populations, implying that variant frequency at <19% may not be clinically relevant. This finding was supported by other deep sequencing studies investigating the role of low frequency variants from clinical isolates obtained from patients on treatment, concluding that they could not be used to predict resistance and inform treatment decisions (Shin et al., 2018; Nimmo et al., 2020a).

The PK properties of BDQ, a long half-life drug, may also explain emerging resistance to BDQ. BDQ in combination with other short half-life drugs like RIF or INH would result in PK mismatch leading to BDQ monotherapy thereby contributing to the acquisition of resistance (Nguyen et al., 2016). As a result of the long terminal elimination half-life of BDQ (5.5 months) the drug remains in plasma even after BDQ cessation since its killing effect is time-dependent (Andries et al., 2005). Drug-drug interaction studies showed RIF and efavirenz reduced the steady-state concentrations of BDQ by 79 and 52%, respectively, thus combining these drugs should be avoided (Svensson et al., 2014).

Primary BDQ resistance, i.e., BDQ resistance prior to any BDQ exposure, has also been reported in patients due to *Rv0678* mutations (Trauner et al., 2017; De Vos et al., 2019; Ghodousi et al., 2019; Peretokina et al., 2020). Published reports demonstrate baseline *Rv0678* variants in 5.4 to 6.6% of patients despite absence of elevated BDQ MIC’s. However, patients harboring these variants documented worse outcomes than patients without baseline *Rv0678* variants (Villellas et al., 2017; Nimmo et al., 2020b). Emerging *Rv0678* variants were detected in 5.7% of patients and was associated with an 8-fold increase in BDQ MIC and worse treatment outcomes compared to those who did not have *Rv0678* variants (Nimmo et al., 2020b). Similar results were documented in a phase II study assessing BDQ efficacy (NCT00910871) with emerging resistance detected in 4.4% of patients (12/205) linked to *Rv0678* variants with >4-fold BDQ MIC increase occurring at week 24 or later in the course of treatment (Pym et al., 2016). Despite this, these patients documented treatment success. While there is a clear role for phenotypic testing during BDQ treatment in order to detect emerging resistance, the detection of low-level baseline *Rv0678* variants is likewise important because of documented worse treatment outcomes (Ghodousi et al., 2019; Polsfuss et al., 2019). However, baseline *Rv0678* variants require more sensitive genotypic methods as they are unlikely to display increased MICs.

Results posted on the preprint server bioRxiv demonstrated that the emergence of *Rv0678* variants pre-dated the use of BDQ/CFZ and some have been detected as early as the 18th century (Van Dorp et al., 2020).^2^ This study found that *Mtb* strains can be naturally resistant to BDQ/CFZ. Further, *Rv0678* variants do not severely impact the fitness cost of the strain but are easily selected for in the presence or absence of drug pressure (Van Dorp et al., 2020). Data suggests the expression of *Rv0678* variants amongst patients even without prior exposure to the drugs, and may in part explain emergence of BDQ/CFZ resistance during treatment (Trauner et al., 2017; Veziris et al., 2017; Villellas et al., 2017; Chawla et al., 2018; Ismail et al., 2018a; Ghajavand et al., 2019a; Polsfuss et al., 2019; Peretokina et al., 2020).

It has recently been demonstrated that the initial acquisition of *Rv0678* variants in clinical isolates confer low to intermediate levels of BDQ resistance. The subsequent additional acquisition and dominance of the *atpE* mutations confers high-level BDQ resistance (Zimenkov et al., 2017; Ismail et al., 2018a; Peretokina et al., 2020). Peretokina et al. (2020) recently elucidated novel double mutations in the *Rv0678* and *atpE* genes amongst BDQ-exposed patients. Isolates from two patients initially harbored *Rv0678* frameshift mutations with low MICs, but subsequent acquisition of A63P in *atpE* led to high level BDQ resistance (MICs of 0.5 and >1 mg/L in 7H11, and 2.0 and >8 mg/L in MGIT). In the third patient a different amino acid change was observed on the same codon (A63V) in *atpE* with an L142R mutation in *Rv0678* (MIC = 1 mg/L on 7H11 and MGIT MIC of >8 mg/L). Zimenkov et al. (2017) similarly observed the emergence of the A63V mutation in *atpE* in addition to L142R mutation in *Rv0678* after BDQ treatment (MIC of 1.0 mg/L in 7H11). Ismail et al. (2018a) demonstrated double mutations in *Rv0678* and *Rv1979c* (mainly G1226A) also observed in BDQ exposed patients; however the *Rv1979c* gene is only implicated in CFZ resistance. This phenomenon was also observed *in vitro* with serial passaging of PZA resistant laboratory-generated mutants exposed to increasing BDQ concentrations. Initially a number of dynamic low frequency *Rv0678* variants appear and disappear, thereafter succeeding organisms appeared with high frequency fixed *atpE* mutations (Ismail et al., 2019a).

The use of modern algorithms and machine-learning tools to assess the effect of *atpE* mutations on BDQ binding showed that clinically identified resistance-conferring mutations (D28A/G/P/V, A63M/P/V, E61D, L59V, and I66M) were localized at the drug binding site or a resistance hotspot (Karmakar et al., 2019). This, however, remains to be performed for *Rv0678* and *pepQ* gene mutations. Peretokina et al. (2020) identified variants in the *Rv0678* gene conferring high-level BDQ resistance: L40S, A99V, L114P, and L142H (7H11 MIC of 0.25 mg/L) and E113K (7H11 MIC of 0.5 mg/L), and assessed hotspot regions (nucleotides 139, 141, and 191) using data from 20 isolates. Additional potential hotspot regions at codons 47 and after 48 in *Rv0678* need investigation, given that frameshift mutations at codons 47 and 48 were observed in other XDR-TB isolates (Martinez et al., 2018; Mokrousov et al., 2019). Frameshift mutations inactivate the repressor activity of *Rv0678* leading to the overexpression of efflux pump activity, expulsion of BDQ out of the *Mtb* cell and thus, elevated BDQ MICs and resistance to the drug (Andries et al., 2014; Peretokina et al., 2020).

#### Clofazimine

Clofazimine was traditionally used to treat leprosy but was repurposed to treat DR-TB (WHO, 2016). It is a key constituent of the new standard short-course regimen for DR-TB treatment as a core second-line agent and is categorized as a WHO group B drug (WHO, 2019b). The drug belongs to the riminophenazine chemical class and is thought to release reactive oxygen species after reduction by NADH dehydrogenase and re-oxidation (Lechartier and Cole, 2015). Previous studies showed that CFZ use was associated with favorable treatment outcomes, net culture conversion and decreased risk of death in MDR- and XDR-TB patients, including HIV co-infected XDR-TB (Gopal et al., 2013; Padayatchi et al., 2014; Pietersen et al., 2014). In contrast, an individual patient data meta-analysis reported no improvement in treatment outcomes in patients on CFZ compared to those who were not treated with CFZ (Fox et al., 2017). Successful treatment outcomes with the inclusion of CFZ in the Bangladesh/WHO recommended short-course MDR-TB regimen renewed interest in the drug (Tang et al., 2015; Duan et al., 2019; Lange et al., 2019; Du et al., 2020). Given its widespread use, mechanisms mediating resistance to the drug and phenotypic criteria defining resistance needs to be established. WHO has recommended an interim CC for CFZ of 1.0 mg/L in MGIT only (WHO, 2018a). As in the case of BDQ, resistance to CFZ is mediated through the acquisition of mutations in the *Rv0678* gene leading to efflux extrusion and in the *pepQ* gene, a putative cytoplasmic peptidase (Hartkoorn et al., 2014; Zhang S. et al., 2015; Ismail et al., 2018a, 2019b). In addition, mutations in the *Rv1979c* gene, encoding a putative permease, may be linked to CFZ resistance (Zhang S. et al., 2015). However, mutations in *pepQ* and *Rv1979c* remain to be fully elucidated. Mutations in *Rv0678* has been associated with low-level resistance to BDQ and CFZ, *pepQ* mutations result in a low-level increase in BDQ and CFZ MICs and mutations in *Rv1979c* confers resistance to CFZ only (Andries et al., 2014; Hartkoorn et al., 2014; Ismail et al., 2019a).

A number of studies reported a diverse range of mutations occurring in the *Rv0678* gene linked to CFZ/BDQ cross-resistance. Hartkoorn et al. (2014) reported CFZ-resistant mutants were associated with cross-resistance to BDQ (4- to 8-fold increased MIC) linked to mutations in *Rv0678* (R134STOP and S63R) (Andries et al., 2014). Ismail et al. (2019b) used laboratory generated mutants to establish the effect of *Rv0678* mutations and cross resistance between BDQ and CFZ. They demonstrated that CFZ-resistant mutants displayed elevated BDQ MIC’s (8 mg/L in MGIT) and BDQ-resistant mutants displayed elevated CFZ MIC’s (≥4 mg/L in MGIT) linked to the S63R/G and R135G variants in *Rv0678*. This implies that *Rv0678* mutations detected will compromise the effect of both drugs. Zhang S. et al. (2015) described 28 novel diverse mutations in the *Rv0678* gene in addition to two previously described mutations (193 G insertion and A202G). Of these, 70% were InDels resulting in frameshift mutations. The 193 G insertion was among the most common mutations detected, previously described in BDQ-resistant patients during MDR-TB treatment, providing further evidence for CFZ/BDQ cross-resistance (Villellas et al., 2017).

Pang et al. (2017) reported resistance to CFZ and BDQ was observed in 5.6 and 3.3% of the XDR-TB isolates, respectively. In this study, patients with CFZ-resistant isolates documented >4 fold increases in BDQ MIC’s linked to the S53P/L and Y157D *Rv0678* mutations (Pang et al., 2017). Polsfuss et al. (2019) reported on the emergence of an *Rv0678* (185ins_CAG) mutation in an XDR-TB patient undergoing treatment with CFZ [0.25 mg/L MIC in the resazurin microtiter assay (REMA)] which also conferred BDQ resistance. In contrast, Xu et al. (2017) reported on 5 CFZ resistant pre- and XDR-TB clinical isolates with no prior exposure to BDQ or CFZ. Four isolates had different *Rv0678* mutations (M146T, S2I, S53L, and L117R) associated with cross-resistance to BDQ [with CFZ MIC’s of >1.2 mg/L using the microplate alamarBlue assay (MABA)]. Whilst a strong correlation between CFZ and BDQ resistance due to *Rv0678* variants has been demonstrated, Ismail et al. (2018a) reported that this association is incomplete. BDQ resistance emanating from *Rv0678* variants conferred 100% cross resistance to CFZ; however, CFZ resistance linked to *Rv0678* conferred only a third cross resistance to BDQ. In contrast, Kadura et al. (2020) systematic review demonstrated full cross-resistance between CFZ and BDQ resulting from mutations in *Rv0678* and *pepQ* stressing the importance of BDQ and CFZ phenotypic testing. These studies reiterate the intrinsic role that *Rv0678* mutations play in a cross-resistance mechanism and that exposure to either CFZ or BDQ can compromise the subsequent use of the other.

Almeida et al. (2016) described the role of loss of function *pepQ* mutations in conferring low-level BDQ resistance with cross-resistance to CFZ. *PepQ* mutants were selected in mice through BDQ treatment with and without CFZ, resulting in a 4-fold increase in MIC for both drugs. Subsequent treatment with efflux pump inhibitors resulted in a marked decrease in MIC. Three loss of function mutations (L44P, A14 frameshift, R271 frameshift) resulted in complete disruption of the active site of the gene leading to modest increases in BDQ and CFZ MICs. Zhang S. et al. (2015) identified an E89STOP mutation in CFZ-resistant laboratory-generated mutants that inactivated the function of the cytoplasmic peptidase protein. Liu et al. (2021) reported the first *pepQ* mutation in a CFZ-resistant clinical isolate from an MDR-TB patient receiving BDQ treatment. The L145I mutation resulted in a CFZ MIC of 1 mg/L.

In contrast to mutations in *Rv0678* and *pepQ*, mutations in the *Rv1979c* gene are not linked to BDQ cross-resistance. Zhang S. et al. (2015) first reported the T1052C (V351A) *rv1979c* gene mutation in CFZ-resistant laboratory-generated mutants. Xu et al. (2017) subsequently reported the V52G mutation in *rv1979c* (4-fold increased MIC) from a patient with no prior exposure to both BDQ and CFZ. It can therefore be implied that *Mtb* in clinical isolates can also harbor *rv1979c* mutations with or without prior BDQ/CFZ treatment.

#### Delamanid and Pretomanid

Delamanid (Deltyba™; previously known as OPC-67683) and PRT (previously known as PA-824) are nitroimidazole pro-drugs that are activated by de-azaflavin-dependent nitroreductase (cofactor F420; ddn) encoded by the *ddn* gene (Manjunatha et al., 2006). Following the synthesis of cofactor F420 by a group of enzymes encoded by the genes *fdg1*, *fbiA, fbiB*, and *fbiC*, DLM, first synthesized in 2006, underwent various clinical trials before being approved for MDR-TB treatment in 2014 (Matsumoto et al., 2006; Sasaki et al., 2006; WHO, 2014). It primarily acts to inhibit methoxy mycolic and keto-mycolic acid, integral components of the mycobacterial cell wall (Matsumoto et al., 2006; Lewis and Sloan, 2015). Under aerobic conditions of actively replicating *Mtb*, nitroimidazoles prevents mycolic acid synthesis integral for forming bacterial cell walls (Manjunatha et al., 2009). Under anaerobic conditions, they cease respiration by releasing toxic nitric oxide characterized by reduced intracellular ATP levels (Manjunatha et al., 2009).

Resistance to PRT was associated with mutations in *ddn* and *fgd1* genes and in genes of the F420 biosynthetic pathway (*fbiA, fbiB*, and *fbiC*). H37Rv mutants bearing these mutations were associated with PRT MIC’s ranging between 0.015–0.25 mg/L (Stover et al., 2000; Choi et al., 2001, 2002; Gurumurthy et al., 2013). DLM resistance is associated with the same PRT resistance mutations since both drugs share a common activation pathway. DLM resistance however, occurs at a lower MIC range of 0.006–0.024 mg/L (Matsumoto et al., 2006; Fujiwara et al., 2018). Hence, cross-resistance between DLM and PRT exists, however evidence suggests that this cross-resistance is incomplete (Hurdle et al., 2008). DLM displayed enhanced sterilizing activity compared to PRT against MDR- and XDR-TB isolates with 4-fold lower MIC values (0.016 vs. 0.063 mg/L in MABA), suggesting that MICs should be determined separately for these two nitroimidazoles (Wen et al., 2019). Furthermore, a site directed mutagenesis model showed that the drugs bind differently to the active site of the *ddn* gene (Lee et al., 2020a). There are a number of conflicting reports on the CC value for DLM. Otsuka proposed a CC for DLM at 0.2 mg/L in Middlebrook 7H10/11 while WHO recommended an interim CC of 0.016 mg/L (Middlebrook 7H11) and 0.06 mg/L (MGIT) (Stinson et al., 2016; WHO, 2018a). Moreover, the EUCAST suggests a CC of 0.06 mg/L (Middlebrook 7H10/7H11) for DLM (EUCAST, 2020). CC values for PRT remain to established.

Bloemberg et al. (2015) reported acquired DLM resistance associated with *fgd1* (G49 frameshift) and *fbiA* (D49T) gene mutations in an XDR-TB isolate from a patient exposed to DLM leading to high-level resistance. Hoffmann et al. (2016) reported the same *fbiA* D49Y mutation and the R175H mutation following DLM therapy. Schena et al. (2016) reported the occurrence of various termination mutations in *ddn* (W88STOP; *n* = 3 MDR isolates) and *fbiA* (K250STOP; *n* = 1 XDR isolate) mutations in patients with no prior exposure to DLM. Another mutation, G104S, in *fgd1* was reported in pre-XDR-TB clinical isolate after 3 months of DLM treatment (Ghodousi et al., 2019). More recently, Wen et al. (2019) documented the E249K *fbiA* mutation with high-level DLM resistance in MDR- and XDR-TB clinical isolates.

Yang et al. (2018) reported two novel *ddn* mutations G81D (*n* = 2 isolates with MIC > 1.6 mg/L) and G81S (*n* = 31 isolates with MIC range of 0.4 −>1.6 mg/L) in MDR- and XDR-TB clinical isolates. However, it was not known whether or not patients were exposed to DLM. Emerging low-level DLM resistance, together with BDQ and CFZ resistance, was observed in an XDR-TB isolate (Polsfuss et al., 2019). The novel G53D *ddn* mutation was associated with increased DLM MIC of 0.25 mg/L in REMA and the variant frequency increased from 78 to 100% over time. Fujiwara et al. (2018) also reported the L107P *ddn* mutation (DLM MIC of 1 mg/L in 7H11) and 14 amino acid deletion (codons 59–101; DLM MIC of >8 mg/L) in MDR-TB clinical isolates with no prior exposure to DLM. These studies suggest the natural occurrence of *ddn* mutations, without prior nitroimidazole exposure and warrants further investigation.

In the case of the *fbiC* gene, Pang et al. (2017) reported the V318I mutation from in two XDR-TB patients displaying DLM resistance associated with an MIC of 32 mg/L. Two recent studies described the emergence of DLM resistance mutations in DLM naïve patients. Nimmo et al. (2020a) reported the emergence of *fbiC* A487 frameshift and S534STOP mutations at low frequencies in DR-TB patients following 6 months of treatment. Reichmuth et al. (2020) reported the A416V and Y678G *fbiC* mutations with associated low-level DLM resistance and other *fbiC* mutations: c1161t, c1680t, g-11a, a-32g, D375N, I128V, K571E, and A505T. The *fbiC* R322L and N336K mutations detected in PRT-resistant laboratory-generated mutants, contributed to more than half the PRT resistance *in vitro*. However their role in clinical isolates remain unelucidated (Rifat et al., 2020).

Pretomanid resistance was attributed to the detection of resistance mutations in PRT laboratory-generated mutants in five non-essential genes, contributing to varying levels resistance; *ddn*: 29%, *fbiC*: 26%, *fbiA*: 19%, *fgd1*: 7%, *fbiB*: 2%, as was similarly demonstrated elsewhere (Haver et al., 2015; Fujiwara et al., 2018). However, 17% of PRT resistant mutants harbored no mutations in these genes suggesting alternate mechanisms of resistance (Haver et al., 2015). More recently, a novel resistance mechanism to DLM and PRT not previously associated with nitroimidazole resistance was described by Rifat et al. (2020). The *fbiD* gene (*rv2983* – phosphoenol-pyruvate guanylyl transferase) was associated with 9% of nitroimidazole resistance in PRT resistant laboratory-generated mutants. The study also demonstrated that spontaneous PRT resistant mutations were naturally existent, and can also be selectively induced by treatment with PRT. The contribution of the *fbiC* mutations accounted for 56% of PRT resistance and the novel *fbiD* gene mutations accounted for 17% of resistance (Haver et al., 2015; Rifat et al., 2020). Mutations in the *fbiD* gene may account for unexplained PRT resistance, however, cross-resistance to DLM remains to be identified. Interestingly, a majority of the *fbiD* and *fbiB* mutants exhibited high-level PRT resistance and only intermediate level DLM resistance. Similarly DLM resistance was less common in mutants compared to PRT resistance (Lee et al., 2020a; Rifat et al., 2020). Reichmuth et al. (2020) recently investigated several DLM resistance mutations from global clinical isolates naïve to nitroimidazoles. MIC’s of DLM resistant isolates ranged from 0.015 −>8 mg/L in broth micro dilution (BMD). Interestingly, they reported a rare *fbiB* mutation (T302I) and novel *fbiD* mutation (A21T and A300G) in clinical isolates.

#### Linezolid

Linezolid is an oxazolidinone that acts by binding to the V domain of the 50S ribosomal subunit, specifically the peptidyl-transferase center, thereby inhibiting early protein synthesis. High- and low-level resistance to LZD has been associated with mutations in the *rrl* (23S rRNA) and *rplC* (encodes ribosomal protein L3) genes, respectively (Hillemann et al., 2008; Beckert et al., 2012). The *rplV* and *rplD* genes encoding ribosomal protein L4 and L22 were initially thought to be involved in LZD resistance, but the *rplC* gene was described as a novel mechanism of resistance to LZD in MRSA and subsequently detected in *Mtb* (Richter et al., 2007; Beckert et al., 2012). No mutations were detected in the other described targets. LZD was originally used to treat MRSA infections and was re-purposed for MDR and XDR-TB treatment (Locke et al., 2009). An individual patient meta-analysis of 12 030 MDR-TB patients from 50 datasets found that LZD use (alongside other drugs) was associated with reduced mortality and treatment success (Ahmad et al., 2018). WHO reaffirmed the CC of 1 mg/L in MGIT and recommended an interim CC of 1.0 mg/L in Middlebrook 7H10 and 7H11 (WHO, 2018a).

Mutations in the *rrl* gene was initially considered the most significant mechanism of LZD resistance reported to be widely distributed across the gene with four predominant mutations (G2229T, G2270T/C, G2746A, and G2814T). Early *in vitro* studies by Hillemann et al. (2008) reported high-level LZD resistance associated with the G2061T (also reported as G2299T) mutation (*n* = 4; MIC of 32 mg/L) and a G2576T (also reported as G2814T) mutation (*n* = 1; MIC of 16 mg/L) in the *rrl* gene in LZD-resistant mutants of *Mtb.* However, 5 additional LZD-resistant mutants with an MIC range of 4–8 mg/L displayed no mutations in the *rrl* gene. Similar results were obtained by McNeil et al. (2017) in laboratory-generated LZD-resistant mutants. Zhang et al. (2014) subsequently reported the G2061T mutation in two clinical isolates associated with an MIC of 32 mg/L; one of which had additional mutation in *rplC* (T460/C154R). Two studies reported the G2576T and A2572C *rrl* double mutants in XDR-TB patients associated with an MIC of 4 mg/L (Bloemberg et al., 2015; Somoskovi et al., 2015). Zimenkov et al. (2017) reported the occurrence of the *rrl* G2576T and G2294A single mutations in two LZD resistant isolates. Lee et al. (2012) reported four cases of acquired LZD resistance with *rrl* mutations (*n* = 1; G2576T with MIC of 4 mg/L in 2 isolates of the patient) (*n* = 1; G2447T with MIC of 16 mg/L) and two cases with *rplC* mutations (described below). The *rrl* G2270T/C mutation, initially selected in laboratory-generated LZD-resistant mutants, was subsequently reported in clinical isolates by Wasserman et al. (Balasubramanian et al., 2014; Zhang et al., 2016). The G2270T was detected in two clinical isolates with an MIC of 2 and 4 mg/L. In addition they also reported the G2576T *rrl* mutation (*n* = 4; G2576T with MIC of 4–8 mg/L) and the G2810C (*n* = 1; no available MIC) (Wasserman et al., 2019). The G2746A has not been reported in clinical isolates (Balasubramanian et al., 2014; Zhang et al., 2016).

*rplC* gene mutations are emerging as a significant mechanism of LZD resistance in *Mtb* with the T460C as the most dominant mutation associated with an MIC range of 4–8 mg/L and in clinical isolates from two XDR-TB patients. Beckert et al. (2012) was the first to report the role of the T460C mutation in the *rplC* gene and its association with LZD resistance in LZD resistant *Mtb* mutants selected *in vitro* (MIC of 4–8 mg/L) and in two clinical isolates. This finding was supported by a number of *in vitro* studies confirming the role of *rplC* mutations (Balasubramanian et al., 2014; Makafe et al., 2016; Zhang et al., 2016; McNeil et al., 2017). Subsequently the mutation was observed as the dominant LZD resistance mechanism in clinical isolates from DR-TB patients. Lee et al. (2012) described two cases of acquired LZD resistance in subsequent isolates from patients (*n* = 3; T460C/C154R with MIC range of 0.5–4 mg/L). Similarly, Perdigão et al. (2016) reported a cased of acquired low-level linezolid resistance associated with this mutation. Two studies subsequently reported emerging LZD resistance in patients undergoing treatment with a LZD containing regimen. Zimenkov et al. (2017) reported the *rplC* T460C mutation in nine isolates obtained from 27 patients failing treatment. This was associated with a change in MIC from 0.5–1 mg/L in pre-treatment isolates to 4–8 mg/L in LZD resistant isolates bearing the mutation. Wasserman et al. (2019) reported the same mutation in 11 isolates associated with an MIC range of 4–8 mg/L. In contrast to laboratory-generated mutants, MICs detected in clinical isolates were found to be lower, 0.5–8 mg/L compared to 4–8 mg/L, respectively (Lee et al., 2012; Perdigão et al., 2016; Zimenkov et al., 2017; Wasserman et al., 2019).

### Detection of Mixed Infection and Its Impact on Diagnosis and Treatment in Tuberculosis

Tuberculosis is generally caused by infection of a single strain of *Mtb*, however, the advent of advanced molecular genotyping methods and deep sequencing technology has demonstrated that an individual could be infected with multiple genetically distinct strains, referred to as mixed infection. Prior to these advances, it was assumed that TB disease resulted from infection with a single *Mtb* strain and initial infection provides a measure of protection against infection with a secondary strain. The complexity of mixed bacterial populations has significant implications on an individual and public health level (Rinder, 2001; Rinder et al., 2001; Cohen et al., 2012). Mixed infection with strains of varying resistance profiles could confound phenotypic and genotypic diagnostics for detection of drug resistance. This in turn impacts treatment selection, treatment success and could drive the selection of DR sub-populations during treatment (Zetola et al., 2014a,b; Zheng et al., 2015; Baffoe-Bonnie et al., 2019). At a public health level, mixed infection could result in an underestimation of the ongoing transmission of *Mtb*. In the event that only one strain from a patient harboring mixed infection is transmitted to a secondary patient, transmission may not be inferred if the same strain was not identified in the index case.

Early epidemiological studies demonstrated the prevalence of mixed infection is higher in high TB-incidence settings, where infection pressure and transmission rates are higher (Tarashi et al., 2017). Prevalence of mixed infection is reported to be between 10–20% in high incidence settings (Warren et al., 2003; Cohen et al., 2012; Sobkowiak et al., 2018; Vargas et al., 2021). A recent retrospective analysis compared rates of mixed infection in geographically distinct countries in Africa and outside Africa. This was based on the rationale that countries in Africa carry a high burden of HIV-associated TB, high rates TB incidence and transmission. Thus, HIV-TB co-infected individuals with a compromised immune response in such settings would result in an increased prevalence of mixed infections. However, there was no difference in the prevalence observed in African countries compared with countries outside Africa. The reported prevalence ranged from 2.3 to 19% in Africa compared to <0.4 to 15.7% in the countries outside of Africa (McIvor et al., 2017).

Historically, mixed infection was detected using traditional genotyping methods, such as spoligotyping, *IS6110* restriction fragment length polymorphism (RFLP), and Mycobacterial Interspersed Repetitive-Unit variable-number tandem repeat (MIRU-VNTR) (Mathema et al., 2006; Pholwat et al., 2013; Naidoo and Dookie, 2018). However, given the limited sensitivity of these methods, as an abundance of at least 10% of the minor strain is required for detection, the estimated rate based on mathematical modeling is much higher (Cohen et al., 2012). Deep WGS techniques provide the ultimate resolution for typing of *Mtb* strains. SNPs detected by mapping sequencing reads to a reference genome are used to infer the genomic distance between *Mtb* isolates (Walker et al., 2013; Nikolayevskyy et al., 2016; Naidoo and Dookie, 2018; Diel et al., 2019). Clinical *Mtb* isolates from solid/liquid cultures are comprised of a mixture of bacterial colonies emanating from the original sputum, thus deep sequencing data of such samples contains information referring to the genetic diversity of the within host bacterial population (Trauner et al., 2017; Shockey et al., 2019; Nimmo et al., 2020a). Since *Mtb* is haploid in nature, the presence of a large number of high-quality heterogeneous SNPs suggests potential mixed infection. However, due to interference of sequencing error from deep sequencing, identification of high-quality heterogeneous SNPs requires an abundance of at least 30% of the minor strain in a mixed infection (Black et al., 2015). However, mixed infection can only be identified with certainty if the infection is driven by genetically distinct strains with a SNP difference of at least 100 (Guerra-Assunca̧õ et al., 2015a). Mixed infection involving < 5 SNPs could indicate microevolution after infection (relapse) rather than mixed infection (or reinfection) (Bryant et al., 2013; Witney et al., 2017). An intermediary SNP difference ranging between 5 and 100 can be explained by relapse in post-treatment isolates from a minority genotype undetected in pre-treatment isolates (Witney et al., 2017). Various rates of mixed infection have been reported using different techniques in the range of 0.4 to 57.1%. The rates of mixed infection were reported to be in the range of 3.4–14.1% using phage typing, 0.4–18.5% using IS6110-RFLP, 0.6–57.1% using spoligotyping, 0.6–55.95% using MIRU-VNTR, and 3.3–12.7% by using WGS (Tarashi et al., 2017). These studies confirm the prevalence of mixed infection, however, the frequency of detection varies depending on study design, sample size and genotyping technique.

Additional challenges complicating detection of mixed infection by genotyping is the variation in results obtained when using direct clinical samples compared to culture as the starting material as well as the use of a single sample vs. multiple samples. Early studies utilized an array of samples including multiple pre-treatment cultures as well as multiple isolates collected serially over time (Hanekom et al., 2013). Comparison of the frequency of mixed infection among these studies indicate an increased likelihood of detecting mixed infection compared to a single sputum sample. Increased odds of detecting mixed infection also occurs with inclusion of multiple samples from different lung cavities compared to a single sample representative of upper airway secretions (Tarashi et al., 2017). Furthermore, use of clinical specimens eliminates culture selection bias and reveals true composition of the bacterial population. Nimmo et al. (2019) recently demonstrated decreased genetic diversity amongst TB isolates from culture compared to direct sputum samples. The study demonstrated a loss of genetic diversity and selection of *Mtb* subpopulations in culture whereas the clonal diversity in paired sputum was much greater.

There is a paucity of data on the impact of mixed infection on TB treatment outcomes. A limited number of studies have reported that the presence of mixed-strain infections involving a combination of DR and susceptible strains results in poor treatment outcomes (Theisen et al., 1995; van Rie et al., 1999; Niemann et al., 2000; Baldeviano-Vidalon et al., 2005; Kamakoli et al., 2017). Additionally, it has been well documented that different strains of *Mtb* elicit varying immune responses which impacts disease pathology and mortality. However, interaction between multiple strains and the host immune system in the case of mixed infection remains to be elucidated (McIvor et al., 2017). From a diagnostic perspective, the selection of only one strain from a mixed-strain infection during culture could potentially underestimate drug resistance and result in sub-optimal treatment and selection of further resistance.

## Discussion

DR-TB remains a significant impediment to TB control programs globally. In the last 5 years, the DR-TB and diagnostic landscape has evolved significantly. The introduction of new drugs and novel combinations has led to the first ever short-course injection free treatment for the most severe forms of the disease. In line with the changes to programmatic treatment and management guidance, WHO has subsequently revised their hierarchical grouping of anti-TB drugs and have recently proposed updated definitions of pre-XDR and XDR-TB incorporating resistance to BDQ and LZD. Pre-XDR TB is now defined as strains that fulfill the definition of RR-TB/MDR-TB with additional resistance to any fluoroquinolone. XDR-TB is now defined strains that fulfill the definition of RR-TB/MDR-TB with additional resistance to any fluoroquinolone and BDQ or LZD. In fulfilment of the End TB strategy goals, access to comprehensive DST and early diagnosis of all individuals with TB is a key priority.

Despite the documented limitations associated with WGS, the technology for *Mtb* has progressed from research laboratories to clinical care and public health applications in well-resourced settings such as the United Kingdom and Europe (Pankhurst et al., 2016; Cabibbe et al., 2018). WGS has become the standard of care in these settings, replacing phenotypic testing for prediction of susceptibility to all first-line drugs creating a pathway for individualized treatment approaches. The greatest impact of WGS in resource limited settings is its potential role in individualized treatment in patients with resistance to second line TB drugs. Individualized treatment could significantly contain resistance amplification and overcome use of sub-optimal treatment regimens, impacting the trajectory of the DR-TB epidemic. Therapy tailored to the patient will be highly beneficial in areas where HIV-TB co-infection is prevalent or mixed TB infections are common. Nevertheless, the applicability of WGS-based individualized treatment in low-income settings is hindered by the cost and complexity of the platform and lack of expertise required for bioinformatics analysis. Interventions or new molecular assays specifically identifying mixed infections are greatly needed in high TB-burden areas.

Whilst the studies reviewed here have greatly improved our understanding of the recent advances linked to direct and targeted sputum-based WGS technology, the mechanisms of resistance to new/repurposed drugs and the effect of mixed infections in TB treatment, it highlights significant challenges that remain. Lack of standardized CC’s and breakpoint values for DLM/PRT impede advancements to determine other gene regions potentially driving resistance to the nitroimidazoles. Currently, no commercial genotypic assay covers resistance detection against the nitroimidazoles given that the molecular mechanisms mediating their resistance are not completely understood. The wide-spread introduction of new drugs in the absence of standardized DST has led to rapid emergence of drug resistance. This review highlights apparent gaps in our knowledge of the mechanisms contributing to resistance for these new drugs. This underscores the need for a combination of genotypic and phenotypic techniques to monitor treatment response to curb emerging resistance and further dissemination drug resistance.

## Author Contributions

ND, AK, and KN contributed to the conception and design of the work. AK and ND acquired most of the information and wrote sections and AK wrote, edited and formatted the first and final draft. KN critically appraised the literature for important intellectual content. All authors contributed to manuscript revision, read and approved the submitted version for final publication.

## Conflict of Interest

The authors declare that the research was conducted in the absence of any commercial or financial relationships that could be construed as a potential conflict of interest.

## Publisher’s Note

All claims expressed in this article are solely those of the authors and do not necessarily represent those of their affiliated organizations, or those of the publisher, the editors and the reviewers. Any product that may be evaluated in this article, or claim that may be made by its manufacturer, is not guaranteed or endorsed by the publisher.

## Footnotes

1
https://www.biorxiv.org/content/10.1101/2021.04.08.438862v3.full.pdf

2
https://www.biorxiv.org/content/10.1101/2020.10.06.328799v1

## Abbreviations

Mtb: *Mycobacterium tuberculosis*.

## References

[B1] Abrahams K. A. Besra G. S. (2018). Mycobacterial cell wall biosynthesis: a multifaceted antibiotic target. *Parasitology* 145 116–133. 10.1017/S0031182016002377 27976597 PMC5964476

[B2] Achkar S. El, Demanche C. Osman M. Rafei R. Ismail M. B. (2019). Drug-Resistant Tuberculosis, Lebanon, 2016 - 2017. *Emerg. Infect. Dis.* 25 2016–2017. 10.3201/eid2503.181375 30789124 PMC6390733

[B3] Ahmad N. Ahuja S. D. Akkerman O. W. Alffenaar J. W. C. Anderson L. F. Baghaei P. (2018). Treatment correlates of successful outcomes in pulmonary multidrug-resistant tuberculosis: an individual patient data meta-analysis. *Lancet* 392 821–834. 10.1016/S0140-6736(18)31644-31641 30215381 PMC6463280

[B4] Allix-Béguec C. Arandjelovic I. Bi L. Beckert P. Bonnet M. Bradley P. (2018). Prediction of susceptibility to first-line tuberculosis drugs by DNA sequencing. *N. Engl. J. Med.* 379 1403–1415. 10.1056/NEJMoa1800474 30280646 PMC6121966

[B5] Almeida D. Ioerger T. Tyagi S. Li S. Y. Mdluli K. Andries K. (2016). Mutations in pepQ confer low-level resistance to bedaquiline and clofazimine in *Mycobacterium tuberculosis*. *Antimicrob. Agents Chemother.* 60 4590–4599. 10.1128/AAC.00753-716 27185800 PMC4958187

[B6] Al-Saeedi M. Al-Hajoj S. (2017). Diversity and evolution of drug resistance mechanisms in *Mycobacterium tuberculosis*. *Infect Drug Resist* 10 333–342. 10.2147/IDR.S144446 29075131 PMC5648319

[B7] Andres S. Merker M. Heyckendorf J. Kalsdorf B. Rumetshofer R. Indra A. (2020). Bedaquiline-Resistant tuberculosis: dark clouds on the horizon. *Am. J. Respir. Crit. Care Med.* 201 1564–1568. 10.1164/rccm.201909-1819LE 32053752

[B8] Andries K. Verhasselt P. Guillemont J. Göhlmann H. W. H. Neefs J. M. Winkler H. (2005). A diarylquinoline drug active on the ATP synthase of *Mycobacterium tuberculosis*. *Science* 307 223–227. 10.1126/science.1106753 15591164

[B9] Andries K. Villellas C. Coeck N. Thys K. Gevers T. Vranckx L. (2014). Acquired resistance of *Mycobacterium tuberculosis* to bedaquiline. *PLoS One* 9:e102135. 10.1371/journal.pone.0102135 25010492 PMC4092087

[B10] Auld S. C. Shah N. S. Mathema B. Brown T. S. Ismail N. Omar S. V. (2018). Extensively drug-resistant tuberculosis in South Africa: genomic evidence supporting transmission in communities. *Eur. Respir. J.* 52:1800246. 10.1183/13993003.00246-2018 30115614 PMC6195447

[B11] Baffoe-Bonnie A. Houpt E. R. Turner L. Dodge D. Heysell S. K. (2019). Drug-Susceptible and multidrug-resistant mycobacterium tuberculosis in a single patient. *Emerg. Infect. Dis.* 25 2120–2121. 10.3201/eid2511.180638 31454310 PMC6810186

[B12] Balasubramanian V. Solapure S. Iyer H. Ghosh A. Sharma S. Kaur P. (2014). Bactericidal activity and mechanism of action of AZD5847, a novel oxazolidinone for treatment of tuberculosis. *Antimicrob. Agents Chemother.* 58 495–502. 10.1128/AAC.01903-1913 24189255 PMC3910779

[B13] Baldeviano-Vidalon G. Quispe-Torres N. Bonilla-Asalde C. Gastiaburu-Rodriguez D. Pro-Cuba J. Llanos-Zavalga F. (2005). Multiple infection with resistant and sensitive *M. tuberculosis* strains during treatment of pulmonary tuberculosis patients. *Int. J. Tuberc Lung Dis.* 9 1155–1160. 16229228

[B14] Battaglia S. Spitaleri A. Cabibbe A. M. Meehan C. J. Utpatel C. Ismail N. (2020). Characterization of genomic variants associated with resistance to bedaquiline and delamanid in naive mycobacterium tuberculosis clinical strains. *J. Clin. Microbiol.* 58 1304–1324. 10.1128/JCM.01304-1320 PMC7587096 32907992

[B15] Beckert P. Hillemann D. Kohl T. A. Kalinowski J. Richter E. Niemann S. (2012). rplC T460C identified as a dominant mutation in linezolid-resistant Mycobacterium tuberculosis strains. *Antimicrob. Agents Chemother.* 56 2743–2745. 10.1128/AAC.06227-6211 22371899 PMC3346602

[B16] Black P. A. de Vos M. Louw G. E. van der Merwe R. G. Dippenaar A. Streicher E. M. (2015). Whole genome sequencing reveals genomic heterogeneity and antibiotic purification in *Mycobacterium tuberculosis* isolates. *BMC Genomics* 16:857. 10.1186/s12864-015-2067-2062 26496891 PMC4619333

[B17] Bloemberg G. Gagneux S. Bottger E. C. (2015). Acquired resistance to bedaquiline and delamanid in therapy for Tuberculosis. *N. Engl. J. Med.* 373 1986–1988. 10.1056/NEJMc1505196 26559594 PMC4681277

[B18] Bouzouita I. Cabibbe A. M. Trovato A. Draoui H. Ghariani A. Midouni B. (2018). Is sequencing better than phenotypic tests for the detection of pyrazinamide resistance? *Int. J. Tuberc Lung Dis.* 22 661–666. 10.5588/ijtld.17.0715 29862951

[B19] Bradley P. Gordon N. C. Walker T. M. Dunn L. Heys S. Huang B. (2015). Rapid antibiotic-resistance predictions from genome sequence data for Staphylococcus aureus and *Mycobacterium tuberculosis*. *Nat. Commun.* 6:10063. 10.1038/ncomms10063 26686880 PMC4703848

[B20] Brimacombe M. Hazbon M. Motiwala A. S. Alland D. (2007). Antibiotic resistance and single-nucleotide polymorphism cluster grouping type in a multinational sample of resistant *Mycobacterium tuberculosis* isolates. *Antimicrob. Agents Chemother.* 51 4157–4159. 10.1128/AAC.00619-617 17846140 PMC2151444

[B21] Brosch R. Gordon S. Marmiesse M. Brodin P. Buchrieser C. Eiglmeier K. (2002). A new evolutionary scenario for the *Mycobacterium tuberculosis* complex. *Proc. Natl. Acad. Sci. U S A.* 99 3684–3689. 10.1073/pnas.052548299 11891304 PMC122584

[B22] Brown A. C. Bryant J. M. Einer-Jensen K. Holdstock J. Houniet D. T. Chan J. Z. M. (2015). Rapid whole-genome sequencing of mycobacterium tuberculosis isolates directly from clinical samples. *J. Clin. Microbiol.* 53 2230–2237. 10.1128/JCM.00486-415 25972414 PMC4473240

[B23] Bryant J. M. Harris S. R. Parkhill J. Dawson R. Diacon A. H. van Helden P. (2013). Whole-genome sequencing to establish relapse or re-infection with *Mycobacterium tuberculosis*: a retrospective observational study. *Lancet Respir Med.* 1 786–792. 10.1016/S2213-2600(13)70231-70235 24461758 PMC3861685

[B24] Cabibbe A. M. Spitaleri A. Battaglia S. Colman R. E. Suresh A. Uplekar S. (2020). Application of targeted next-generation sequencing assay on a portable sequencing platform for culture-free detection of drug-resistant tuberculosis from clinical samples. *J. Clin. Microbiol.* 58 1–9. 10.1128/JCM.00632-620 PMC7512157 32727827

[B25] Cabibbe A. M. Trovato A. De Filippo M. R. Ghodousi A. Rindi L. Garzelli C. (2018). Countrywide implementation of whole genome sequencing: an opportunity to improve tuberculosis management, surveillance and contact tracing in low incidence countries. *Eur. Respir. J.* 51:1800387. 10.1183/13993003.00387-2018 29650560

[B26] Castro R. A. D. Borrell S. Gagneux S. (2020). The within-host evolution of antimicrobial resistance in *Mycobacterium tuberculosis*. *FEMS Microbiol. Rev.* 45:fuaa071. 10.1093/femsre/fuaa071 33320947 PMC8371278

[B27] Chatterjee M. Bhattacharya S. Karak K. Dastidar S. G. (2013). Effects of different methods of decontamination for successful cultivation of *Mycobacterium tuberculosis*. *Indian J. Med. Res.* 138 541–548. 24434262 PMC3868068

[B28] Chawla K. Martinez E. Kumar A. Shenoy V. P. Sintchenko V. (2018). Whole-genome sequencing reveals genetic signature of bedaquiline resistance in a clinical isolate of *Mycobacterium tuberculosis*. *J. Glob Antimicrob Resist* 15 103–104. 10.1016/j.jgar.2018.09.006 30248414

[B29] Chesov D. Lange C. Heyckendorf J. (2019). Molecular-based tuberculosis drug susceptibility testing: one size fits all? *Int. J. Tuberc Lung Dis.* 23 879–880. 10.5588/ijtld.19.0414 31533876

[B30] Chesov E. Chesov D. Maurer F. P. Andres S. Utpatel C. Barilar I. (2021). Emergence of bedaquiline-resistance in a high-burden country of tuberculosis. *Eur. Respir. J.* Online ahead of print. 10.1183/13993003.00621-2021 34503982 PMC8943268

[B31] Choi K. P. Bair T. B. Bae Y. M. Daniels L. (2001). Use of transposon Tn5367 mutagenesis and a nitroimidazopyran-based selection system to demonstrate a requirement for fbiA and fbiB in coenzyme F420 biosynthesis by *Mycobacterium bovis* BCG. *J. Bacteriol.* 183 7058–7066. 10.1128/JB.183.24.7058-7066.2001 11717263 PMC95553

[B32] Choi K. P. Kendrick N. Daniels L. (2002). Demonstration that fbiC is required by *Mycobacterium bovis* BCG for coenzyme F420 and FO biosynthesis. *J. Bacteriol.* 184 2420–2428. 10.1128/JB.184.9.2420-2428.2002 11948155 PMC134996

[B33] Cirillo D. M. Miotto P. Tagliani E. The, and ReSeqTB Consortium. (2016). Reaching consensus on drug resistance conferring mutations (Part 1). *Int. J. Mycobacteriol.* 5 S31–S32. 10.1016/j.ijmyco.2016.09.062 28043595

[B34] Cohen K. A. Abeel T. Manson, McGuire A. Desjardins C. A. Munsamy V. (2015). Evolution of extensively drug-resistant tuberculosis over four decades: whole genome sequencing and dating analysis of *Mycobacterium tuberculosis* isolates from KwaZulu-Natal. *PLoS Med.* 12:e1001880. 10.1371/journal.pmed.1001880 26418737 PMC4587932

[B35] Cohen T. van Helden P. D. Wilson D. Colijn C. McLaughlin M. M. Abubakar I. (2012). Mixed-strain *Mycobacterium tuberculosis* infections and the implications for tuberculosis treatment and control. *Clin. Microbiol. Rev.* 25 708–719. 10.1128/CMR.00021-12 23034327 PMC3485752

[B36] Cole S. T. Brosch R. Parkhill J. Garnier T. Churcher C. Harris D. (1998). Deciphering the biology of mycobacterium tuberculosis from the complete genome sequence. *Nature* 393 537–544. 10.1038/31159 9634230

[B37] Coll F. McNerney R. Preston M. D. Guerra-Assunção J. A. Warry A. Hill-Cawthorne G. (2015). Rapid determination of anti-tuberculosis drug resistance from whole-genome sequences. *Genome Med.* 7:51. 10.1186/s13073-015-0164-160 26019726 PMC4446134

[B38] Colman R. E. Anderson J. Lemmer D. Lehmkuhl E. Georghiou S. B. Heaton H. (2016). Rapid drug susceptibility testing of drug-resistant mycobacterium tuberculosis isolates directly from clinical samples by use of amplicon sequencing: a proof-of-concept study. *J. Clin. Microbiol.* 54 2058–2067. 10.1128/JCM.00535-516 27225403 PMC4963505

[B39] Colman R. E. Mace A. Seifert M. Hetzel J. Mshaiel H. Suresh A. (2019). Whole-genome and targeted sequencing of drug-resistant Mycobacterium tuberculosis on the iSeq100 and MiSeq: a performance, ease-of-use, and cost evaluation. *PLoS Med.* 16:e1002794. 10.1371/journal.pmed.1002794 31039166 PMC6490892

[B40] Colman R. E. Schupp J. M. Hicks N. D. Smith D. E. Buchhagen J. L. Valafar F. (2015). Detection of low-level mixed-population drug resistance in *Mycobacterium tuberculosis* using high fidelity amplicon sequencing. *PLoS One* 10:e0126626. 10.1371/journal.pone.0126626 25970423 PMC4430321

[B41] Conradie F. Diacon A. H. Ngubane N. Howell P. Everitt D. Crook A. M. (2020). Treatment of highly drug-resistant pulmonary tuberculosis. *N. Engl. J. Med.* 382 893–902. 10.1056/NEJMoa1901814 32130813 PMC6955640

[B42] Cox E. Laessig K. (2014). FDA approval of bedaquiline — the benefit-risk balance for drug-resistant tuberculosis. *N. Engl. J. Med.* 371:689. 10.1056/NEJMp1314385 25140952

[B43] Cox H. Hughes J. Black J. Nicol M. P. (2018). Precision medicine for drug-resistant tuberculosis in high-burden countries: is individualised treatment desirable and feasible? *Lancet Infect. Dis.* 18 e282–e287. 10.1016/S1473-3099(18)30104-X 29548923

[B44] Cui Z. Zhou Y. Li H. Zhang Y. Zhang S. Tang S. (2012). Complex sputum microbial composition in patients with pulmonary tuberculosis. *BMC Microbiol.* 12:1. 10.1186/1471-2180-12-276 23176186 PMC3541192

[B45] da Silva P. E. A. Palomino J. C. (2011). Molecular basis and mechanisms of drug resistance in *Mycobacterium tuberculosis*: classical and new drugs. *J. Antimicrob Chemother* 66 1417–1430. 10.1093/jac/dkr173 21558086

[B46] Daum L. T. Rodriguez J. D. Worthy S. A. Ismail N. A. Omar S. V. Dreyer A. W. (2012). Next-generation ion torrent sequencing of drug resistance mutations in *Mycobacterium tuberculosis* strains. *J. Clin. Microbiol.* 50 3831–3837. 10.1128/JCM.01893-1812 22972833 PMC3502959

[B47] De Vos M. Ley S. D. Wiggins K. B. Derendinger B. Dippenaar A. Grobbelaar M. (2019). Bedaquiline microheteroresistance after cessation of tuberculosis treatment. *N. Engl. J. Med.* 380 2178–2180. 10.1056/NEJMc1815121 31141643 PMC6518951

[B48] Derache A. Iwuji C. C. Baisley K. Danaviah S. Marcelin A. Calvez V. (2018). Impact of next generation sequencing defined HIV pre-treatment drug resistance on virological outcomes in the ANRS 12249 treatment as prevention trial. *Clin. Infect. Dis.* 69 207–214. 10.1093/cid/ciy881 30321314 PMC6603266

[B49] Diacon A. H. Donald P. R. Pym A. Grobusch M. Patientia R. F. Mahanyele R. (2012). Randomized pilot trial of eight weeks of bedaquiline (TMC207) treatment for multidrug-resistant tuberculosis: long-term outcome, tolerability, and effect on emergence of drug resistance. *Antimicrob. Agents Chemother.* 56 3271–3276. 10.1128/AAC.06126-6111 22391540 PMC3370813

[B50] Diacon A. H. Pym A. Grobusch M. P. de los Rios J. M. Gotuzzo E. Vasilyeva I. (2014). Multidrug-Resistant tuberculosis and culture conversion with bedaquiline. *N. Engl. J. Med.* 371 723–732. 10.1056/nejmoa1313865 25140958

[B51] Diel R. Kohl T. A. Maurer F. P. Merker M. Walter K. M. Hannemann J. (2019). Accuracy of whole-genome sequencing to determine recent tuberculosis transmission: an 11-year population-based study in Hamburg. *Germany. Eur. Respir. J.* 54 2–5. 10.1183/13993003.01154-2019 31467121 PMC6881715

[B52] Dilliott A. A. Farhan S. M. K. Ghani M. Sato C. Liang E. Zhang M. (2018). Targeted next-generation sequencing and bioinformatics pipeline to evaluate genetic determinants of constitutional disease. *J. Vis. Exp*. 4:57266. 10.3791/57266 29683450 PMC5933375

[B53] Dookie N. Rambaran S. Padayatchi N. Mahomed S. Naidoo K. (2018). Evolution of drug resistance in *Mycobacterium tuberculosis*: a review on the molecular determinants of resistance and implications for personalized care. *J. Antimicrob Chemother* 73 1138–1151. 10.1093/jac/dkx506 29360989 PMC5909630

[B54] Doughty E. L. Sergeant M. J. Adetifa I. Antonio M. Pallen M. J. (2014). Culture-independent detection and characterisation of Mycobacterium tuberculosis and *M. africanum* in sputum samples using shotgun metagenomics on a benchtop sequencer. *PeerJ* 2:e585. 10.7717/peerj.585 25279265 PMC4179564

[B55] Doyle R. M. Burgess C. Williams R. Gorton R. Booth H. Brown J. (2018). Direct whole-genome sequencing of sputum accurately identifies drug-resistant mycobacterium tuberculosis faster than MGIT culture sequencing. *J. Clin. Microbiol.* 56 e666–e618. 10.1128/JCM.00666-618 PMC6062781 29848567

[B56] Du Y. Qiu C. Chen X. Wang J. Jing W. Pan H. (2020). Treatment outcome of a shorter regimen containing clofazimine for multidrug-resistant tuberculosis: a randomized control trial in China. *Clin. Infect. Dis.* 71 1047–1054. 10.1093/cid/ciz915 31549147

[B57] Duan H. Chen X. Li Z. Pang Y. Jing W. Liu P. (2019). Clofazimine improves clinical outcomes in multidrug-resistant tuberculosis: a randomized controlled trial. *Clin. Microbiol. Infect.* 25 190–195. 10.1016/j.cmi.2018.07.012 30036672

[B58] Egli A. Blanc D. S. Greub G. Keller P. M. Lazarevic V. Lebrand A. (2018). Improving the quality and workflow of bacterial genome sequencing and analysis: paving the way for a Switzerland-wide molecular epidemiological surveillance platform. *Swiss Med. Wkly* 148:w14693. 10.4414/smw.2018.14693 30552858

[B59] EUCAST (2020). *The European Committee on Antimicrobial Susceptibility Testing. Breakpoint Tables for Interpretation of MICs and Zone Diameters.* Sweden: EUCAST. Version 10.0, 2020.

[B60] Feuerriegel S. Schleusener V. Beckert P. Kohl T. A. Miotto P. Cirillo D. M. (2015). PhyResSE: a web tool delineating *Mycobacterium tuberculosis* antibiotic resistance and lineage from whole-genome sequencing data. *J. Clin. Microbiol.* 53 1908–1914. 10.1128/JCM.00025-15 25854485 PMC4432036

[B61] Fox G. J. Benedetti A. Cox H. Koh W. Viiklepp P. Ahuja S. (2017). Group 5 drugs for multidrug-resistant tuberculosis: individual patient data meta-analysis. *Eur. Respir. J.* 49:1600993. 10.1183/13993003.00993-2016 28049171

[B62] Fujiwara M. Kawasaki M. Hariguchi N. Liu Y. Matsumoto M. (2018). Mechanisms of resistance to delamanid, a drug for *Mycobacterium tuberculosis*. *Tuberculosis* 108 186–194. 10.1016/j.tube.2017.12.006 29523322

[B63] Genoscreen (2019). *Deeplex^®^Myc-TB From Tuberculosis Clinical Samples to Drug Resistance Profile.* Available online at: https://www.genoscreen.fr/images/genoscreen-advance/Deeplex-technical_note-v05.pdf (accessed December 12, 2019)

[B64] Ghajavand H. Kamakoli M. K. Khanipour S. Dizaji S. P. Masoumi M. Jamnani F. R. (2019a). High prevalence of bedaquiline resistance in treatment-naive tuberculosis patients and verapamil effectiveness. *Antimicrob. Agents Chemother.* 63:e02530-18. 10.1128/AAC.02530-18 30602521 PMC6395892

[B65] Ghajavand H. Kargarpour Kamakoli M. Khanipour S. Pourazar Dizaji S. Masoumi M. Rahimi Jamnani F. (2019b). Scrutinizing the drug resistance mechanism of multi- and extensively-drug resistant *Mycobacterium tuberculosis*: mutations versus efflux pumps. *Antimicrob Resist. Infect. Control* 8:70. 10.1186/s13756-019-0516-514 31073401 PMC6498538

[B66] Ghodousi A. Rizvi A. H. Baloch A. Q. Ghafoor A. Khanzada F. M. Qadir M. (2019). Acquisition of cross-resistance to bedaquiline and clofazimine following treatment for tuberculosis in pakistan. *Antimicrob. Agents Chemother.* 63 1–5. 10.1128/AAC.00915-919 PMC6709449 31262765

[B67] Gilpin C. Korobitsyn A. Weyer K. (2016). Current tools available for the diagnosis of drug-resistant tuberculosis. *Ther. Adv. Infect. Dis.* 3 145–151. 10.1177/2049936116673553 28386407 PMC5375090

[B68] Gopal M. Padayatchi N. Metcalfe J. Z. O’Donnell M. R. (2013). Systematic review of clofazimine for the treatment of drug-resistant tuberculosis. *Int. J. Tuberc. Lung Dis.* 17 1001–1007. 10.5588/ijtld.12.0144 23541151 PMC4003893

[B69] Guerra-Assunca̧õ J. A. Houben R. M. G. J. Crampin A. C. Mzembe T. Mallard K. Coll F. (2015a). Recurrence due to relapse or reinfection with mycobacterium tuberculosis: a whole-genome sequencing approach in a large, population-based cohort with a high HIV infection prevalence and active follow-up. *J. Infect. Dis.* 211 1154–1163. 10.1093/infdis/jiu574 25336729 PMC4354982

[B70] Guerra-Assunca̧õ J. Crampin A. C. Houben R. M. G. J. Mzembe T. Mallard K. Coll F. (2015b). Large-scale whole genome sequencing of *M. tuberculosis* provides insights into transmission in a high prevalence area. *eLife* 4:e05166. 10.7554/eLife.05166 25732036 PMC4384740

[B71] Guglielmetti L. Sougakoff W. Maitre T. Brossier F. Jarlier V. Robert J. (2019). Poor performance of rapid molecular tests to define eligibility for the shortcourse multidrug-resistant tuberculosis regimen. *Clin. Infect. Dis.* 68 1410–1411. 10.1093/cid/ciy808 30239638

[B72] Gurumurthy M. Rao M. Mukherjee T. Rao S. P. S. Boshoff H. I. Dick T. (2013). A novel F 420-dependent anti-oxidant mechanism protects *Mycobacterium tuberculosis* against oxidative stress and bactericidal agents. *Mol. Microbiol.* 4 744–755. 10.1111/mmi.12127 23240649 PMC3567243

[B73] Hain Lifescience GmbH (2021a). *GenoType MTBDRplus VER 2.0.* Available online at: https://www.hain-lifescience.de/en/products/microbiology/mycobacteria/tuberculosis/genotype-mtbdrplus.html (Accessed September 6, 2019)

[B74] Hain Lifescience GmbH (2021b). *GenoType MTBDRsl VER 1.0 and VER 2.0.* Available online at: https://www.hain-lifescience.de/en/products/microbiology/mycobacteria/tuberculosis/genotype-mtbdrsl.html (Accessed September 6, 2019)

[B75] Hanekom M. Streicher E. M. Van de Berg D. Cox H. McDermid C. Bosman M. (2013). Population structure of mixed mycobacterium tuberculosis infection is strain genotype and culture medium dependent. *PLoS One* 8:e70178. 10.1371/journal.pone.0070178 23936157 PMC3728311

[B76] Hartkoorn R. C. Uplekar S. Cole S. T. (2014). Cross-resistance between clofazimine and bedaquiline through upregulation of mmpl5 in mycobacterium tuberculosis. *Antimicrob. Agents Chemother.* 58 2979–2981. 10.1128/AAC.00037-14 24590481 PMC3993252

[B77] Hatherell H. A. Colijn C. Stagg H. R. Jackson C. Winter J. R. Abubakar I. (2016). Interpreting whole genome sequencing for investigating tuberculosis transmission: a systematic review. *BMC Med.* 14:21. 10.1186/s12916-016-0566-x 27005433 PMC4804562

[B78] Haver H. L. Chua A. Ghode P. Lakshminarayana S. B. Singhal A. Mathema B. (2015). Mutations in genes for the F420 biosynthetic pathway and a nitroreductase enzyme are the primary resistance determinants in spontaneous in vitro-selected PA-824-resistant mutants of *Mycobacterium tuberculosis*. *Antimicrob. Agents Chemother.* 59 5316–5323. 10.1128/AAC.00308-315 26100695 PMC4538556

[B79] Hernández P. Punchak M. Camacho M. Hepple P. McNerney R. (2015). Investigating the quality of expectorated sputum for tuberculosis diagnosis in Bolivia. *Int. J. Tuberc. Lung Dis.* 19 1065–1067. 10.5588/ijtld.14.0700 26260825

[B80] Hess J. F. Kohl T. A. Kotrová M. Rönsch K. Paprotka T. Mohr V. (2020). Library preparation for next generation sequencing: a review of automation strategies. *Biotechnol. Adv.* 41:107537. 10.1016/j.biotechadv.2020.107537 32199980

[B81] Heyckendorf J. Andres S. Köser C. U. Olaru I. D. Schön T. Sturegård E. (2018). What is resistance? impact of phenotypic versus molecular drug resistance testing on therapy for multi- and extensively drug-resistant Tuberculosis. *Antimicrob. Agents Chemother.* 62 1–12. 10.1128/AAC.01550-17 29133554 PMC5786814

[B82] Hillemann D. Rüsch-Gerdes S. Richter E. (2008). In vitro-selected linezolid-resistant *Mycobacterium tuberculosis* mutants. *Antimicrob Agents Chemother.* 52 800–801. 10.1128/AAC.01189-07 18070973 PMC2224755

[B83] Hingley-Wilson S. M. Casey R. Connell D. Bremang S. Evans J. T. Hawkey P. M. (2013). Undetected multidrug-resistant tuberculosis amplified by first-line therapy in mixed infection. *Emerg. Infect. Dis.* 19 1138–1141. 10.3201/eid1907.130313 23764343 PMC3713993

[B84] Hoffmann H. Kohl T. A. Hofmann-Thiel S. Merker M. Beckert P. Jaton K. (2016). Delamanid and bedaquiline resistance in mycobacterium tuberculosis ancestral Beijing genotype causing extensively drug-resistant tuberculosis in a tibetan refugee. *Am. J. Respir. Crit. Care Med.* 193 337–340. 10.1164/rccm.201502-0372LE 26829425

[B85] Huitric E. Verhasselt P. Koul A. Andries K. Hoffner S. Andersson D. I. (2010). Rates and mechanisms of resistance development in *Mycobacterium tuberculosis* to a novel diarylquinoline ATP synthase inhibitor. *Antimicrob. Agents Chemother.* 54 1022–1028. 10.1128/AAC.01611-1619 20038615 PMC2825986

[B86] Hunt M. Bradley P. Lapierre S. G. Heys S. Thomsit M. Hall M. B. (2019). Antibiotic resistance prediction for *Mycobacterium tuberculosis* from genome sequence data with mykrobe. *Wellcome Open Res.* 4:191. 10.12688/wellcomeopenres.15603.1 32055708 PMC7004237

[B87] Hurdle J. G. Lee R. B. Budha N. R. Carson E. I. Qi J. Scherman M. S. (2008). A microbiological assessment of novel nitrofuranylamides as anti-tuberculosis agents. *J. Antimicrob Chemother* 62 1037–1045. 10.1093/jac/dkn307 18693235 PMC2566515

[B88] Iketleng T. Lessels R. Dlamini M. T. Mogashoa T. Mupfumi L. Moyo S. (2018). *Mycobacterium tuberculosis* next-generation whole genome sequencing: opportunities and challenges. *Tuberc Res. Treat* 2018:1298542. 10.1155/2018/1298542 30631597 PMC6304523

[B89] Illumina (2020). *History of Sequencing by Synthesis.* San Diego, CA: Illumina.

[B90] ION Torrent Next-Generation Sequencing Technology (2020). *Thermo Fish Sci.* Waltham: Thermofisher.

[B91] Ismail N. A. Omar S. V. Joseph L. Govender N. Blows L. Ismail F. (2018a). Defining bedaquiline susceptibility, resistance, cross-resistance and associated genetic determinants: a retrospective cohort study. *EBioMedicine* 28 136–142. 10.1016/j.ebiom.2018.01.005 29337135 PMC5835552

[B92] Ismail N. Omar S. V. Ismail N. A. Peters R. P. H. (2018b). Collated data of mutation frequencies and associated genetic variants of bedaquiline, clofazimine and linezolid resistance in *Mycobacterium tuberculosis*. *Data Br.* 20 1975–1983. 10.1016/j.dib.2018.09.057 30306102 PMC6172430

[B93] Ismail N. Ismail N. A. Omar S. V. Peters R. P. H. (2019a). In vitro study of stepwise acquisition of rv0678 and atpE mutations conferring bedaquiline resistance. *Antimicrob Agents Chemother* 63 1–6. 10.1128/AAC.00292-19 31138569 PMC6658778

[B94] Ismail N. Peters R. P. H. Ismail N. A. Omar S. V. (2019b). Clofazimine exposure in vitro selects efflux pump mutants and bedaquiline resistance. *Antimicrob Agents Chemother* 63 31–34. 10.1128/AAC.02141-18 30642938 PMC6395909

[B95] Iwai H. Kato-Miyazawa M. Kirikae T. Miyoshi-Akiyama T. (2015). CASTB (the comprehensive analysis server for the Mycobacterium tuberculosis complex): a publicly accessible web server for epidemiological analyses, drug-resistance prediction and phylogenetic comparison of clinical isolates. *Tuberculosis* 95 843–844. 10.1016/j.tube.2015.09.002 26542225

[B96] Kadura S. King N. Nakhoul M. Zhu H. Theron G. Köser C. U. (2020). Systematic review of mutations associated with resistance to the new and repurposed *Mycobacterium tuberculosis* drugs bedaquiline, clofazimine, linezolid, delamanid and pretomanid. *J. Antimicrob Chemother.* 75 2031–2043. 10.1093/jac/dkaa136 32361756 PMC7825472

[B97] Kamakoli M. K. Sadegh H. R. Farmanfarmaei G. Masoumi M. Fateh A. Javadi G. (2017). Evaluation of the impact of polyclonal infection and heteroresistance on treatment of tuberculosis patients. *Sci. Rep.* 7:41410. 10.1038/srep41410 28120910 PMC5264600

[B98] Kaniga K. Aono A. Borroni E. Cirillo D. M. Desmaretz C. Hasan R. (2020). Validation of bedaquiline phenotypic drug susceptibility testing methods and breakpoints: a multilaboratory, multicountry study. *J. Clin. Microbiol.* 58:e01677-19. 10.1128/JCM.01677-1619 31969421 PMC7098739

[B99] Karmakar M. Rodrigues C. H. M. Holt K. E. Dunstan S. J. Denholm J. Ascher D. B. (2019). Empirical ways to identify novel Bedaquiline resistance mutations in AtpE. *PLoS One* 14:e0217169. 10.1371/journal.pone.0217169 31141524 PMC6541270

[B100] Kayomo M. K. Mbula V. N. Aloni M. André E. Rigouts L. Boutachkourt F. (2020). Targeted next-generation sequencing of sputum for diagnosis of drug-resistant TB: results of a national survey in Democratic Republic of the Congo. *Sci. Rep.* 10:10786. 10.1038/s41598-020-67479-67474 32612134 PMC7329841

[B101] Kohl T. A. Utpatel C. Schleusener V. De Filippo M. R. Beckert P. Cirillo D. M. (2018). MTBseq: a comprehensive pipeline for whole genome sequence analysis of Mycobacterium tuberculosis complex isolates. *PeerJ* 6:e5895. 10.7717/peerj.5895 30479891 PMC6238766

[B102] Köser C. U. Cirillo D. M. Miotto P. (2020). How to optimally combine genotypic and phenotypic drug susceptibility testing methods for pyrazinamide. *Antimicrob. Agents Chemother.* 64 e1003–e1020. 10.1128/AAC.01003-1020 PMC7449218 32571824

[B103] Köser C. U. Maurer F. P. Kranzer K. (2019). ‘Those who cannot remember the past are condemned to repeat it’: drug-susceptibility testing for bedaquiline and delamanid. *Int. J. Infect. Dis.* 80 S32–S35. 10.1016/j.ijid.2019.02.027 30818049

[B104] Koul A. Dendouga N. Vergauwen K. Molenberghs B. Vranckx L. Willebrords R. (2007). Diarylquinolines target subunit c of mycobacterial ATP synthase. *Nat. Chem. Biol.* 3 323–324. 10.1038/nchembio884 17496888

[B105] Lam C. Martinez E. Crighton T. Furlong C. Donnan E. Marais B. J. (2021). Value of routine whole genome sequencing for *Mycobacterium tuberculosis* drug resistance detection. *Int. J. Infect. Dis*. 113(Suppl. 1), S48–S54. 10.1016/j.ijid.2021.03.033 33753222

[B106] Lange C. Alghamdi W. A. Al-Shaer M. H. Brighenti S. Diacon A. H. DiNardo A. R. (2018). Perspectives for personalized therapy for patients with multidrug-resistant tuberculosis. *J. Intern. Med.* 284 163–188. 10.1111/joim.12780 29806961

[B107] Lange C. Chesov D. Heyckendorf J. (2019). Clofazimine for the treatment of multidrug-resistant tuberculosis. *Clin. Microbiol. Infect.* 25 128–130. 10.1016/j.cmi.2018.11.010 30472423

[B108] Lechartier B. Cole S. T. (2015). Mode of action of clofazimine and combination therapy with benzothiazinones against *Mycobacterium tuberculosis*. *Antimicrob. Agents Chemother.* 59 4457–4463. 10.1128/AAC.00395-315 25987624 PMC4505229

[B109] Lee M. Lee J. Carroll M. W. Choi H. Min S. Song T. (2012). Linezolid for treatment of chronic extensively drug-resistant Tuberculosis. *N. Engl. J. Med.* 367 1508–1518. 10.1056/nejmoa1201964 23075177 PMC3814175

[B110] Lee R. S. Jean-François Proulx, McIntosh F. Behr M. A. Hanage W. P. (2019). Investigating within-host diversity of *Mycobacterium tuberculosis* reveals novel super-spreaders in the Canadian North. *bioRxiv [preprint]* 10.25557/2310-0435.2019.04.43-49

[B111] Lee B. M. Harold L. K. Almeida D. V. Afriat-Jurnou L. Aung H. L. Forde B. M. (2020a). Predicting nitroimidazole antibiotic resistance mutations in *Mycobacterium tuberculosis* with protein engineering. *PLoS Pathog* 16:e1008287. 10.1371/journal.ppat.1008287 32032366 PMC7032734

[B112] Lee R. S. Proulx J. F. McIntosh F. Behr M. A. Hanage W. P. (2020b). Previously undetected super-spreading of mycobacterium tuberculosis revealed by deep sequencing. *eLife* 9:e53245. 10.7554/eLife.53245 32014110 PMC7012596

[B113] Lewis J. M. Sloan D. J. (2015). The role of delamanid in the treatment of drug-resistant tuberculosis. *Ther. Clin. Risk Manag.* 11 779–791. 10.2147/TCRM.S71076 25999726 PMC4437614

[B114] Ley S. D. de Vos M. Van Rie A. Warren R. M. (2019). Deciphering within-host microevolution of *Mycobacterium tuberculosis* through whole-genome sequencing: the phenotypic impact and way forward. *Microbiol. Mol. Biol. Rev.* 83:e00062-18. 10.1128/mmbr.00062-18 30918049 PMC6684002

[B115] Liu X. Gutacker M. M. Musser J. M. Fu Y. X. (2006). Evidence for recombination in *Mycobacterium tuberculosis*. *J. Bacteriol.* 188 8169–8177. 10.1128/JB.01062-1066 16997954 PMC1698211

[B116] Liu Y. Gao J. Du J. Shu W. Wang L. Wang Y. (2021). Acquisition of clofazimine resistance following bedaquiline treatment for multidrug-resistant tuberculosis. *Int. J. Infect. Dis.* 102 392–396. 10.1016/j.ijid.2020.10.081 33130209

[B117] Locke J. B. Hilgers M. Shaw K. J. (2009). Novel ribosomal mutations in *Staphylococcus aureus* strains identified through selection with the oxazolidinones linezolid and torezolid (TR-700). *Antimicrob. Agents Chemother.* 53 5265–5274. 10.1128/AAC.00871-879 19752277 PMC2786364

[B118] Loman N. J. Constantinidou C. Chan J. Z. M. Halachev M. Sergeant M. Penn C. W. (2012a). High-throughput bacterial genome sequencing: an embarrassment of choice, a world of opportunity. *Nat. Rev. Microbiol.* 10 599–606. 10.1038/nrmicro2850 22864262

[B119] Loman N. J. Misra R. V. Dallman T. J. Constantinidou C. Gharbia S. E. Wain J. (2012b). Performance comparison of benchtop high-throughput sequencing platforms. *Nat. Biotechnol.* 30 434–439. 10.1038/nbt.2198 22522955

[B120] Mahomed S. Naidoo K. Dookie N. Padayatchi N. (2017). Whole genome sequencing for the management of drug-resistant TB in low income high TB burden settings: challenges and implications. *Tuberculosis* 107 137–143. 10.1016/j.tube.2017.09.005 29050762

[B121] Maiga M. Siddiqui S. Diallo S. Diarra B. Traoré B. Shea Y. R. (2012). Failure to recognize nontuberculous mycobacteria leads to misdiagnosis of chronic pulmonary tuberculosis. *PLoS One* 7:e36902. 10.1371/journal.pone.0036902 22615839 PMC3353983

[B122] Makafe G. G. Cao Y. Tan Y. Julius M. Liu Z. Wang C. (2016). Role of the Cys154Arg substitution in ribosomal protein L3 in oxazolidinone resistance in *Mycobacterium tuberculosis*. *Antimicrob. Agents Chemother.* 60 3202–3206. 10.1128/AAC.00152-116 26953211 PMC4862453

[B123] Makhado N. A. Matabane E. Faccin M. Pinçon C. Jouet A. Boutachkourt F. (2018). Outbreak of multidrug-resistant tuberculosis in South Africa undetected by WHO-endorsed commercial tests: an observational study. *Lancet Infect. Dis.* 18 1350–1359. 10.1016/S1473-3099(18)30496-30491 30342828

[B124] Manjunatha U. H. Boshoff H. Dowd C. S. Zhang L. Albert T. J. Norton J. E. (2006). Identification of a nitroimidazo-oxazine-specific protein involved in PA-824 resistance in *Mycobacterium tuberculosis*. *Proc. Natl. Acad. Sci. U S A.* 103 431–436. 10.1073/pnas.0508392103 16387854 PMC1326169

[B125] Manjunatha U. Boshoff H. I. M. Barry C. E. (2009). The mechanism of action of PA-824. *Commun. Integr. Biol.* 2 215–218. 10.4161/cib.2.3.7926 19641733 PMC2717523

[B126] Marin M. G. Vargas R. Harris M. Jeffrey B. Epperson L. E. Durbin D. (2021). Genomic sequence characteristics and the empiric accuracy of short-read sequencing. *bioRxiv [preprint]* 10.1101/2021.04.08.438862

[B127] Martinez E. Hennessy D. Jelfs P. Crighton T. Chen S. C.-A. Sintchenko V. (2018). Mutations associated with in vitro resistance to bedaquiline in *Mycobacterium tuberculosis* isolates in Australia. *Tuberculosis* 111 31–34. 10.1016/j.tube.2018.04.007 30029911

[B128] Mathema B. Kurepina N. E. Bifani P. J. Kreiswirth B. N. (2006). Molecular epidemiology of tuberculosis: current insights. *Clin. Microbiol. Rev.* 19 658–685. 10.1128/CMR.00061-65 17041139 PMC1592690

[B129] Matsumoto M. Hashizume H. Tomishige T. Kawasaki M. Tsubouchi H. Sasaki H. (2006). OPC-67683, a nitro-dihydro-imidazooxazole derivative with promising action against tuberculosis in vitro and in mice. *PLoS Med.* 3:e466. 10.1371/journal.pmed.0030466 17132069 PMC1664607

[B130] McIvor A. Koornhof H. Kana B. D. (2017). Relapse, re-infection and mixed infections in tuberculosis disease. *Pathog Dis.* 75:20. 10.1093/femspd/ftx020 28334088

[B131] McNeil M. B. Dennison D. D. Shelton C. D. Parish T. (2017). In vitro isolation and characterization of oxazolidinone-resistant Mycobacterium tuberculosis. *Antimicrob Agents Chemother* 61:e01296-17. 10.1128/AAC.01296-17 28760892 PMC5610523

[B132] McNerney R. Clark T. G. Campino S. Rodrigues C. Dolinger D. Smith L. (2017). Removing the bottleneck in whole genome sequencing of *Mycobacterium tuberculosis* for rapid drug resistance analysis: a call to action. *Int. J. Infect. Dis.* 56 130–135. 10.1016/j.ijid.2016.11.422 27986491

[B133] McNerney R. Zignol M. Clark T. G. (2018). Use of whole genome sequencing in surveillance of drug resistant tuberculosis. *Expert Rev. Anti Infect. Ther.* 16 433–442. 10.1080/14787210.2018.1472577 29718745

[B134] Meehan C. J. Goig G. A. Kohl T. A. Verboven L. Dippenaar A. Ezewudo M. (2019). Whole genome sequencing of *Mycobacterium tuberculosis*: current standards and open issues. *Nat. Rev. Microbiol.* 17 533–545. 10.1038/s41579-019-0214-215 31209399

[B135] Metcalfe J. Z. Streicher E. Theron G. Colman R. E. Penaloza R. Allender C. (2017). *Mycobacterium tuberculosis* subculture results in loss of potentially clinically relevant heteroresistance. *Antimicrob. Agents Chemother.* 61:e00888-17. 10.1128/AAC.00888-817 28893776 PMC5655066

[B136] Miotto P. Tessema B. Tagliani E. Chindelevitch L. Starks A. M. Emerson C. (2017). A standardised method for interpreting the association between mutations and phenotypic drug resistance in *Mycobacterium tuberculosis*. *Eur. Respir. J.* 50:1701354. 10.1183/13993003.01354-2017 29284687 PMC5898944

[B137] Miotto P. Zhang Y. Cirillo D. M. Yam W. C. (2018). Drug resistance mechanisms and drug susceptibility testing for tuberculosis. *Respirology* 23 1098–1113. 10.1111/resp.13393 30189463

[B138] Mishra H. Reeve B. Palmer Z. Caldwell J. Dolby T. Naidoo C. (2020). Diagnostic accuracy and predictive value of Xpert Ultra and Xpert MTB/RIF for tuberculosis diagnosis in an HIV-endemic setting with a high burden of previous tuberculosis. *Lancet Respir Med.* 8 368–382. 10.1016/S2213-2600(19)30370-30374 32066534

[B139] Mokrousov I. Akhmedova G. Polev D. Molchanov V. Vyazovaya A. (2019). Acquisition of bedaquiline resistance by extensively drug-resistant *Mycobacterium tuberculosis* strain of Central Asian Outbreak clade. *Clin. Microbiol. Infect.* 25 1295–1297. 10.1016/j.cmi.2019.06.014 31229592

[B140] Naidoo K. Dookie N. (2018). Insights into recurrent tuberculosis: relapse versus reinfection and related risk factors. *Tuberculosis* 1–36. 10.5772/intechopen.73601

[B141] Nathavitharana R. R. Cudahy P. G. T. Schumacher S. G. Steingart K. R. Pai M. Denkinger C. M. (2017). Accuracy of line probe assays for the diagnosis of pulmonary and multidrug-resistant tuberculosis: a systematic review and meta-analysis. *Eur. Respir. J.* 49:1601075. 10.1183/13993003.01075-2016 28100546 PMC5898952

[B142] Ndhlovu V. Mandala W. Sloan D. Kamdolozi M. Caws M. Davies G. (2018). Evaluation of the efficacy of two methods for direct extraction of DNA from *Mycobacterium tuberculosis* sputum. *J. Infect. Dev. Ctries* 12 1067–1072. 10.3855/jidc.10592 32027607

[B143] Nguyen T. N. A. Berre V. A. Le, Bañuls A. L. Nguyen T. V. A. (2019). Molecular diagnosis of drug-resistant tuberculosis; a literature review. *Front. Microbiol.* 10:794. 10.3389/fmicb.2019.00794 31057511 PMC6477542

[B144] Nguyen T. V. A. Cao T. B. T. Akkerman O. W. Tiberi S. Vu D. H. Alffenaar J. W. C. (2016). Bedaquiline as part of combination therapy in adults with pulmonary multi-drug resistant tuberculosis. *Expert Rev. Clin. Pharmacol.* 9 1025–1037. 10.1080/17512433.2016.1200462 27322153

[B145] Niemann S. Richter E. Rüsch-Gerdes S. Schlaak M. Greinert U. (2000). Double infection with a resistant and a multidrug-resistant strain of *Mycobacterium tuberculosis*. *Emerg. Infect. Dis.* 6 548–551. 10.3201/eid0605.000518 10998389 PMC2627962

[B146] Nikolayevskyy V. Kranzer K. Niemann S. Drobniewski F. (2016). Whole genome sequencing of *Mycobacterium tuberculosis* for detection of recent transmission and tracing outbreaks: a systematic review. *Tuberculosis* 98 77–85. 10.1016/j.tube.2016.02.009 27156621

[B147] Nimmo C. Brien K. Millard J. Grant A. D. Padayatchi N. Pym A. S. (2020a). Dynamics of within-host *Mycobacterium tuberculosis* diversity and heteroresistance during treatment. *EBioMedicine* 55:102747. 10.1016/j.ebiom.2020.102747 32361247 PMC7195533

[B148] Nimmo C. Millard J. Brien K. Moodley S. van Dorp L. Lutchminarain K. (2020b). Bedaquiline resistance in drug-resistant tuberculosis HIV co-infected patients. *Eur. Respir J.* 55:1902383. 10.1183/13993003.02383-2019 32060065 PMC7270361

[B149] Nimmo C. Shaw L. P. Doyle R. Williams R. Brien K. Burgess C. (2019). Whole genome sequencing *Mycobacterium tuberculosis* directly from sputum identifies more genetic diversity than sequencing from culture. *BMC Genomics* 20:389. 10.1186/s12864-019-5841-5848 31109296 PMC6528373

[B150] Nunn A. J. Phillips P. P. J. Meredith S. K. Chiang C. Y. Conradie F. Dalai D. (2019). A trial of a shorter regimen for rifampin-resistant tuberculosis. *N. Engl. J. Med.* 380 1201–1213. 10.1056/NEJMoa1811867 30865791

[B151] Olayanju O. Limberis J. Esmail A. Oelofse S. Gina P. Pietersen E. (2018). Long-term bedaquiline-related treatment outcomes in patients with extensively drug-resistant tuberculosis from South Africa. *Eur. Respir. J.* 51:1800544. 10.1183/13993003.00544-2018 29700106

[B152] Padayatchi N. Gopal M. Naidoo R. Werner L. Naidoo K. Master I. (2014). Clofazimine in the treatment of extensively drug-resistant tuberculosis with HIV coinfection in South Africa: a retrospective cohort study. *J. Antimicrob Chemother.* 69 3101–3107. 10.1093/jac/dku235 24986495 PMC4195472

[B153] Pang Y. Zong Z. Huo F. Jing W. Ma Y. Dong L. (2017). In vitro drug susceptibility of bedaquiline, delamanid, linezolid, clofazimine, moxifloxacin, and gatifloxacin against extensively drug-resistant tuberculosis in Beijing. China. *Antimicrob Agents Chemother.* 61:e00900-17. 10.1128/AAC.00900-17 28739779 PMC5610515

[B154] Pankhurst L. J. del Ojo, Elias C. Votintseva A. A. Walker T. M. Cole K. (2016). Rapid, comprehensive, and affordable mycobacterial diagnosis with whole-genome sequencing: a prospective study. *Lancet Respir Med.* 4 49–58. 10.1016/S2213-2600(15)00466-X 26669893 PMC4698465

[B155] Perdigão J. Maltez F. Machado D. Silva H. Pereira C. Silva C. (2016). Beyond extensively drug-resistant tuberculosis in Lisbon, Portugal: a case of linezolid resistance acquisition presenting as an iliopsoas abscess. *Int. J. Antimicrob. Agents* 48 569–570. 10.1016/j.ijantimicag.2016.07.026 27769708

[B156] Peretokina I. Krylova L. Y. Antonova O. Kholina M. S. Kulagina E. V. Nosova E. Y. (2020). Reduced susceptibility and resistance to bedaquiline in clinical *M. tuberculosis isolates*. *J. Infect.* 80 527–535. 10.1016/j.jinf.2020.01.007 31981638

[B157] Phelan J. E. O’Sullivan D. M. Machado D. Ramos J. Oppong Y. E. A. Campino S. (2019). Integrating informatics tools and portable sequencing technology for rapid detection of resistance to anti-tuberculous drugs. *Genome Med.* 11:41. 10.1186/s13073-019-0650-x 31234910 PMC6591855

[B158] Pholwat S. Stroup S. Foongladda S. Houpt E. (2013). Digital PCR to detect and quantify heteroresistance in drug resistant *Mycobacterium tuberculosis*. *PLoS One* 8:e57238. 10.1371/journal.pone.0057238 23468945 PMC3584134

[B159] Pietersen E. Ignatius E. Streicher E. M. Mastrapa B. Padanilam X. Pooran A. (2014). Long-term outcomes of patients with extensively drug-resistant tuberculosis in South Africa: a cohort study. *Lancet* 383 1230–1239. 10.1016/S0140-6736(13)62675-62676 24439237

[B160] Polsfuss S. Hofmann-Thiel S. Merker M. Krieger D. Niemann S. Rüssmann H. (2019). Emergence of low-level delamanid and bedaquiline resistance during extremely drug-resistant Tuberculosis treatment. *Clin. Infect. Dis.* 69 1229–1231. 10.1093/cid/ciz074 30933266

[B161] Preiss L. Langer J. D. Yildiz Ö Eckhardt-Strelau L. Guillemont J. E. G. Koul A. (2015). Structure of the mycobacterial ATP synthase Fo rotor ring in complex with the anti-TB drug bedaquiline. *Sci. Adv.* 1:e1500106. 10.1126/sciadv.1500106 26601184 PMC4640650

[B162] Pym A. S. Diacon A. H. Tang S. J. Conradie F. Danilovits M. Chuchottaworn C. (2016). Bedaquiline in the treatment of multidrug- and extensively drug-resistant tuberculosis. *Eur. Respir. J.* 47 564–574. 10.1183/13993003.00724-2015 26647431

[B163] Qian J. Chen R. Wang H. Zhang X. (2020). Role of the PE/PPE family in host-pathogen interactions and prospects for anti-tuberculosis vaccine and diagnostic tool design. *Front. Cell Infect. Microbiol.* 10:594288. 10.3389/FCIMB.2020.594288 33324577 PMC7726347

[B164] Reichmuth M. L. Hömke R. Zürcher K. Sander P. Avihingsanon A. Collantes J. (2020). Natural polymorphisms in mycobacterium tuberculosis conferring resistance to delamanid in drug-naive patients. *Antimicrob. Agents Chemother.* 64 1–5. 10.1128/AAC.00513-20 32868333 PMC7577131

[B165] Richter E. Rüsch-Gerdes S. Hillemann D. (2007). First linezolid-resistant clinical isolates of *Mycobacterium tuberculosis*. *Antimicrob. Agents Chemother.* 51 1534–1536. 10.1128/AAC.01113-1116 17242139 PMC1855508

[B166] Rifat D. Li S.-Y. Ioerger T. Shah K. Lanoix J.-P. Lee J. (2020). Mutations in fbiD (Rv2983) as a novel determinant of resistance to pretomanid and delamanid in *Mycobacterium tuberculosis*. *Antimicrob. Agents Chemother.* 65:e01948-20. 10.1128/aac.01948-1920 33077652 PMC7927868

[B167] Rinder H. (2001). Hetero-resistance: an under-recognised confounder in diagnosis and therapy? *J. Med. Microbiol.* 50 1018–1020. 10.1099/0022-1317-50-12-1018 11761184

[B168] Rinder H. Mieskes K. T. Löscher T. (2001). Heteroresistance in *Mycobacterium tuberculosis*. *Int. J. Tuberc. Lung Dis.* 5 339–345. 11334252

[B169] Sasaki H. Haraguchi Y. Itotani M. Kuroda H. Hashizume H. Tomishige T. (2006). Synthesis and antituberculosis activity of a novel series of optically active 6-nitro-2,3-dihydroimidazo[2,1-b]oxazoles. *J. Med. Chem.* 49 7854–7860. 10.1021/jm060957y 17181168

[B170] Satta G. Lipman M. Smith G. P. Arnold C. Kon O. M. McHugh T. D. (2018). Mycobacterium tuberculosis and whole-genome sequencing: how close are we to unleashing its full potential? *Clin. Microbiol. Infect.* 24 604–609. 10.1016/j.cmi.2017.10.030 29108952

[B171] Schena E. Nedialkova L. Borroni E. Battaglia S. Cabibbe A. M. Niemann S. (2016). Delamanid susceptibility testing of *Mycobacterium tuberculosis* using the resazurin microtitre assay and the BACTECTM MGITTM960 system. *J. Antimicrob Chemother.* 71 1532–1539. 10.1093/jac/dkw044 27076101

[B172] Schleusener V. Köser C. U. Beckert P. Niemann S. Feuerriegel S. (2017). *Mycobacterium tuberculosis* resistance prediction and lineage classification from genome sequencing: comparison of automated analysis tools. *Sci. Rep.* 7:46327. 10.1038/srep46327 28425484 PMC7365310

[B173] Schnippel K. Ndjeka N. Maartens G. Meintjes G. Master I. Ismail N. (2018). Effect of bedaquiline on mortality in South African patients with drug-resistant tuberculosis: a retrospective cohort study. *Lancet Respir. Med.* 6 699–706. 10.1016/S2213-2600(18)30235-30232 30001994

[B174] Schumacher S. G. Sohn H. Qin Z. Z. Gore G. Davis J. L. Denkinger C. M. (2016). Impact of molecular diagnostics for tuberculosis on patient-important outcomes: a systematic review of study methodologies. *PLoS One* 11:e0151073. 10.1371/JOURNAL.PONE.0151073 26954678 PMC4783056

[B175] Seid A. Berhane N. (2021). Molecular mechanisms of genetic interaction (Epistasis) in the evolution and management of antibiotic resistance tuberculosis: current consequence and future perspectives. *Int. J. Pathog Res.* 6 58–70. 10.9734/ijpr/2021/v6i230160

[B176] Shah N. S. Auld S. C. Brust J. C. M. Mathema B. Ismail N. Moodley P. (2017). Transmission of extensively drug-resistant Tuberculosis in South Africa. *N. Engl. J. Med.* 376 243–253. 10.1056/NEJMoa1604544 28099825 PMC5330208

[B177] Shin S. S. Modongo C. Baik Y. Allender C. Lemmer D. Colman R. E. (2018). Mixed *Mycobacterium tuberculosis*-strain infections are associated with poor treatment outcomes among patients with newly diagnosed Tuberculosis. Independent of Pretreatment Heteroresistance. *J. Infect. Dis.* 218 1974–1982. 10.1093/infdis/jiy480 30085153 PMC6217728

[B178] Shockey A. C. Dabney J. Pepperell C. S. (2019). Effects of host, sample, and in vitro culture on genomic diversity of pathogenic mycobacteria. *Front. Genet.* 10:477. 10.3389/fgene.2019.00477 31214242 PMC6558051

[B179] Siddiqi S. H. Rüsch-Gerdes S. (2006). *MGITTM Procedure Manual.* Available online at: https://www.finddx.org/wp-content/uploads/2016/02/mgit_manual_nov2006.pdf (accessed January 27, 2021)

[B180] Sobkowiak B. Glynn J. R. Houben R. M. G. J. Mallard K. Phelan J. E. Guerra-Assunção J. A. (2018). Identifying mixed *Mycobacterium tuberculosis* infections from whole genome sequence data. *BMC Genomics* 19:613. 10.1186/s12864-018-4988-z 30107785 PMC6092779

[B181] Somoskovi A. Bruderer V. Hömke R. Bloemberg G. V. Böttger E. C. (2015). A mutation associated with clofazimine and bedaquiline cross-resistance in MDR-TB following bedaquiline treatment. *Eur. Respir. J.* 45 554–557. 10.1183/09031936.00142914 25359333

[B182] Sreevatsan S. Pan X. I. Stockbauer K. E. Connell N. D. Kreiswirth B. N. Whittam T. S. (1997). Restricted structural gene polymorphism in the *Mycobacterium tuberculosis* complex indicates evolutionarily recent global dissemination. *Proc. Natl. Acad. Sci. U S A.* 94 9869–9874. 10.1073/pnas.94.18.9869 9275218 PMC23284

[B183] Starks A. M. Aviles E. Cirillo D. M. Denkinger C. M. Dolinger D. L. Emerson C. (2015). Collaborative effort for a centralized worldwide Tuberculosis relational sequencing data platform. *Clin. Infect. Dis.* 61 S141–S146. 10.1093/cid/civ610 26409275 PMC4583571

[B184] Steiner A. Stucki D. Coscolla M. Borrell S. Gagneux S. (2014). KvarQ: targeted and direct variant calling from fastq reads of bacterial genomes. *BMC Genomics* 15:881. 10.1186/1471-2164-15-881 25297886 PMC4197298

[B185] Stinson K. W. Eisenach K. Kayes S. Matsumoto M. Siddiqi S. Nakashima S. (2014). *Mycobacteriology Laboratory Manual - Global Laboratory Initiative Advancing TB Diagnosis.* Available online at: https://www.who.int/tb/laboratory/mycobacteriology-laboratory-manual.pdf (Accessed January 27, 2021)

[B186] Stinson K. Kurepina N. Venter A. Fujiwara M. Kawasaki M. Timm J. (2016). MIC of delamanid (OPC-67683) against *Mycobacterium tuberculosis* clinical isolates and a proposed critical concentration. *Antimicrob. Agents Chemother.* 60 3316–3322. 10.1128/AAC.03014-3015 26976868 PMC4879369

[B187] Stover C. K. Warrener P. VanDevanter D. R. Sherman D. R. Arain T. M. Langhorne M. H. (2000). A small-molecule nitroimidazopyran drug candidate for the treatment of tuberculosis. *Nature* 405 962–966. 10.1038/35016103 10879539

[B188] Supply P. Warren R. M. Bañuls A.-L. Lesjean S. Van Der Spuy G. D. Lewis L.-A. (2003). Linkage disequilibrium between minisatellite loci supports clonal evolution of *Mycobacterium tuberculosis* in a high tuberculosis incidence area. *Mol. Microbiol.* 47 529–538. 10.1046/j.1365-2958.2003.03315.x 12519202

[B189] Svensson E. M. Murray S. Karlsson M. O. Dooley K. E. (2014). Rifampicin and rifapentine significantly reduce concentrations of bedaquiline, a new anti-TB drug. *J. Antimicrob Chemother.* 70 1106–1114. 10.1093/jac/dku504 25535219 PMC4356204

[B190] Tafess K. Ng T. T. L. Lao H. Y. Leung K. S. S. Tam K. K. G. Rajwani R. (2019). Targeted sequencing workflows for comprehensive drug resistance profiling of mycobacterium tuberculosis cultures using illumina miseq and nanopore MinION: comparison of analytical and diagnostic performance, turnaround time and cost. *bioRxiv [preprint]* 10.1101/760462 32402055

[B191] Tagliani E. Cabibbe A. M. Miotto P. Borroni E. Toro J. C. Mansjö M. (2015). Diagnostic performance of the new version (v2.0) of GenoType MTBDRsl assay for detection of resistance to fluoroquinolones and second-line injectable drugs: a multicenter study. *J. Clin. Microbiol.* 53 2961–2969. 10.1128/JCM.01257-1215 26179309 PMC4540937

[B192] Tagliani E. Cirillo D. M. Ködmön C. van der Werf M. J. (2018). EUSeqMyTB to set standards and build capacity for whole genome sequencing for tuberculosis in the EU. *Lancet Infect. Dis.* 18:377. 10.1016/S1473-3099(18)30132-30134 29582760

[B193] Tagliani E. Hassan M. O. Waberi Y. Filippo M. R. De, Falzon D. (2017). Culture and Next-generation sequencing-based drug susceptibility testing unveil high levels of drug-resistant-TB in Djibouti: results from the first national survey. *Sci. Rep.* 7:17672. 10.1038/s41598-017-17705-17703 29247181 PMC5732159

[B194] Tang S. Yao L. Hao X. Liu Y. Zeng L. Liu G. (2015). Clofazimine for the treatment of multidrug-resistant tuberculosis: prospective, multicenter, randomized controlled study in China. *Clin. Infect. Dis.* 60 1361–1367. 10.1093/cid/civ027 25605283

[B195] Tarashi S. Fateh A. Mirsaeidi M. Siadat S. D. Vaziri F. (2017). Mixed infections in tuberculosis: the missing part in a puzzle. *Tuberculosis* 107 168–174. 10.1016/j.tube.2017.09.004 29050766

[B196] Theisen A. Reichel C. Rüsch-Gerdes S. Haas W. H. Rockstroh J. K. Spengler U. (1995). Mixed-strain infection with a drug-sensitive and multidrug-resistant strain of *Mycobacterium tuberculosis*. *Lancet* 345 1512–1513. 10.1016/S0140-6736(95)91073-91075 7769926

[B197] Trauner A. Liu Q. Via L. E. Liu X. Ruan X. Liang L. (2017). The within-host population dynamics of *Mycobacterium tuberculosis* vary with treatment efficacy. *Genome Biol.* 18:71. 10.1186/s13059-017-1196-1190 28424085 PMC5395877

[B198] Tweed C. D. Dawson R. Burger D. A. Conradie A. Crook A. M. Mendel C. M. (2019). Bedaquiline, moxifloxacin, pretomanid, and pyrazinamide during the first 8 weeks of treatment of patients with drug-susceptible or drug-resistant pulmonary tuberculosis: a multicentre, open-label, partially randomised, phase 2b trial. *Lancet Respir Med.* 7 1048–1058. 10.1016/S2213-2600(19)30366-30362 31732485 PMC7641992

[B199] Van Dorp L. Nimmo C. Torres Ortiz A. Pang J. Acman M. Tan C. C. S. (2020). Detection of a bedaquiline/clofazimine resistance reservoir in *Mycobacterium tuberculosis* predating the antibiotic era. *bioRxiv [preprint]* 10.1101/2020.10.06.328799

[B200] van Rie A. Warren R. M. Richardson M. Victor T. C. Gie R. P. Enarson D. A. (1999). Exogenous reinfection as a cause of recurrent Tuberculosis after curative treatment. *N. Engl. J. Med.* 341 1174–1179. 10.1056/NEJM199910143411602 10519895

[B201] Vargas R. Freschi L. Marin M. Epperson L. E. Smith M. Oussenko I. (2021). In-host population dynamics of *Mycobacterium tuberculosis* complex during active disease. *eLife* 10:e61805. 10.7554/eLife.61805 33522489 PMC7884073

[B202] Veziris N. Bernard C. Guglielmetti L. Le, Du D. Marigot-Outtandy D. (2017). Rapid emergence of *Mycobacterium tuberculosis* bedaquiline resistance: lessons to avoid repeating past errors. *Eur. Respir. J.* 49 1–11. 10.1183/13993003.01719-2016 28182568

[B203] Villellas C. Coeck N. Meehan C. J. Lounis N. de Jong B. Rigouts L. (2017). Unexpected high prevalence of resistance-associated Rv0678 variants in MDR-TB patients without documented prior use of clofazimine or bedaquiline. *J. Antimicrob Chemother.* 72 684–690. 10.1093/jac/dkw502 28031270 PMC5400087

[B204] Votintseva A. A. Bradley P. Pankhurst L. Al E. (2017a). Same-Day diagnostic and surveillance data for tuberculosis via whole-genome sequencing of direct respiratory samples. *J. Clin. Microbiol.* 55 1285–1298. 10.1128/JCM 28275074 PMC5405248

[B205] Votintseva A. A. Bradley P. Pankhurst L. Del Ojo, Elias C. Loose M. (2017b). Same-day diagnostic and surveillance data for tuberculosis via whole-genome sequencing of direct respiratory samples. *J. Clin. Microbiol.* 55 1285–1298. 10.1128/JCM.02483-2416 28275074 PMC5405248

[B206] Votintseva A. A. Pankhurst L. J. Anson L. W. Morgan M. R. Gascoyne-Binzi D. Walker T. M. (2015). Mycobacterial DNA extraction for whole-genome sequencing from early positive liquid (MGIT) cultures. *J. Clin. Microbiol.* 53 1137–1143. 10.1128/JCM.03073-3014 25631807 PMC4365189

[B207] Walker T. M. Cruz A. L. G. Peto T. E. Smith E. G. Esmail H. Crook D. W. (2017). Tuberculosis is changing. *Lancet Infect. Dis.* 17 359–361. 10.1016/S1473-3099(17)30123-30128 28298254

[B208] Walker T. M. Ip C. L. C. Harrell R. H. Evans J. T. Kapatai G. Dedicoat M. J. (2013). Whole-genome sequencing to delineate *Mycobacterium tuberculosis* outbreaks: a retrospective observational study. *Lancet Infect. Dis.* 13 137–146. 10.1016/S1473-3099(12)70277-70273 23158499 PMC3556524

[B209] Walker T. M. Kohl T. A. Omar S. V. Hedge J. Del Ojo, Elias C. (2015). Whole-genome sequencing for prediction of *Mycobacterium tuberculosis* drug susceptibility and resistance: a retrospective cohort study. *Lancet Infect. Dis.* 15 1193–1202. 10.1016/S1473-3099(15)00062-66 26116186 PMC4579482

[B210] Walker T. M. Lalor M. K. Broda A. Ortega L. S. Parker L. Churchill S. (2014). Assessment of *Mycobacterium tuberculosis* transmission in Oxfordshire, UK, 2007-12, with whole pathogen genome sequences: an observational study. *Lancet Respir Med.* 2 285–292. 10.1016/S2213-2600(14)70027-X 24717625 PMC4571080

[B211] Walter K. S. Colijn C. Cohen T. Mathema B. Liu Q. Bowers J. (2020). Genomic variant-identification methods may alter *Mycobacterium tuberculosis* transmission inferences. *Microb Genomics* 6:mgen000418. 10.1099/mgen.0.000418 32735210 PMC7641424

[B212] Warren R. M. Victor T. C. Streicher E. M. Richardson M. Beyers N. Gey Van Pittius N. C. (2003). Patients with active tuberculosis often have different strains in the same sputum specimen. *Am. J. Respir. Crit. Care Med.* 169 610–614. 10.1164/rccm.200305-714OC 14701710

[B213] Wasserman S. Louw G. Ramangoaela L. Barber G. Hayes C. Omar S. V. (2019). Linezolid resistance in patients with drug-resistant TB and treatment failure in South Africa. *J. Antimicrob Chemother.* 74 2377–2384. 10.1093/jac/dkz206 31081017 PMC6640298

[B214] Wen S. Jing W. Zhang T. Zong Z. Xue Y. Shang Y. (2019). Comparison of in vitro activity of the nitroimidazoles delamanid and pretomanid against multidrug-resistant and extensively drug-resistant tuberculosis. *Eur. J. Clin. Microbiol. Infect. Dis.* 38 1293–1296. 10.1007/s10096-019-03551-w 30953211

[B215] Whitfield M. G. Soeters H. M. Warren R. M. York T. Sampson S. L. Streicher E. M. (2015). A global perspective on pyrazinamide resistance: systematic review and meta-analysis. *PLoS One* 10:e0133869. 10.1371/journal.pone.0133869 26218737 PMC4517823

[B216] WHO (2013). *Automated Real-time Nucleic Acid Amplification Technology for Rapid and Simultaneous Detection of Tuberculosis and Rifampicin Resistance: Xpert MTB/RIF Assay for the Diagnosis of Pulmonary and Extrapulmonary TB in Adults and Children.* Geneva: WHO. 25473701

[B217] WHO (2014). *The use of Delamanid in the Treatment of Multidrug-resistant Tuberculosis. Interim Policy Guidance.* Geneva: WHO. 26110189

[B218] WHO (2016). *WHO Treatment Guidelines for Drug-resistant Tuberculosis 2016 Update.* Geneva: WHO. 27748093

[B219] WHO (2018a). *Technical Report on Critical Concentrations for Drug Susceptibility Testing of Medicines Used in the Treatment of Drug-resistant Tuberculosis.* Geneva: WHO.

[B220] WHO (2018b). *The use of Next-generation Sequencing Technologies for the Detection of Mutations Associated with Drug Resistance in Mycobacterium Tuberculosis Complex: Technical Guide.* Geneva: WHO.

[B221] WHO (2019a). *Rapid Communication: Key Changes to the Treatment of Drug-resistant Tuberculosis.* Geneva: WHO.

[B222] WHO (2019b). *WHO Consolidated Guidelines on Drug-resistant Tuberculosis Treatment.* Geneva: WHO. 30946559

[B223] WHO (2020a). *Global Tuberculosis Report 2020.* Geneva: WHO.

[B224] WHO (2020b). *WHO Consolidated Guidelines on Tuberculosis. Module 4: Treatment - Drug-Resistant Tuberculosis Treatment.* Geneva: WHO. 32603040

[B225] WHO (2021a). *Catalogue of Mutations in Mycobacterium tuberculosis Complex and Their Association with Drug Resistance.* Geneva: WHO.

[B226] WHO (2021b). *Meeting Report of the WHO Expert Consultation on the Definition of Extensively Drug-resistant Tuberculosis, 27-29 October 2020.* Geneva: WHO.

[B227] Witney A. A. Bateson A. L. E. Jindani A. Phillips P. P. J. Coleman D. Stoker N. G. (2017). Use of whole-genome sequencing to distinguish relapse from reinfection in a completed tuberculosis clinical trial. *BMC Med.* 15:71. 10.1186/s12916-017-0834-834 28351427 PMC5371199

[B228] Witney A. A. Gould K. A. Arnold A. Coleman D. Delgado R. Dhillon J. (2015). Clinical application of whole-genome sequencing to inform treatment for multidrug-resistant tuberculosis cases. *J. Clin. Microbiol.* 53 1473–1483. 10.1128/jcm.02993-2914 25673793 PMC4400773

[B229] Xu J. Wang B. Hu M. Huo F. Guo S. Jing W. (2017). Primary clofazimine and bedaquiline resistance among isolates from patients with multidrug-resistant tuberculosis. *Antimicrob. Agents Chemother.* 61 1–8. 10.1128/AAC.00239-217 PMC5444180 28320727

[B230] Yadon A. N. Maharaj K. Adamson J. H. Lai Y. P. Sacchettini J. C. Ioerger T. R. (2017). A comprehensive characterization of PncA polymorphisms that confer resistance to pyrazinamide. *Nat. Commun.* 8:588. 10.1038/s41467-017-00721-722 28928454 PMC5605632

[B231] Yang J. S. Kim K. J. Choi H. Lee S. H. (2018). Delamanid, bedaquiline, and linezolid minimum inhibitory concentration distributions and resistance-related gene mutations in multidrug-resistant and extensively drug-resistant tuberculosis in Korea. *Ann. Lab. Med.* 38 563–568. 10.3343/alm.2018.38.6.563 30027700 PMC6056398

[B232] Zetola N. M. Modongo C. Moonan P. K. Ncube R. Matlhagela K. Sepako E. (2014a). Clinical outcomes among persons with pulmonary tuberculosis caused by *Mycobacterium tuberculosis* isolates with phenotypic heterogeneity in results of drug-susceptibility tests. *J. Infect. Dis.* 209 1754–1763. 10.1093/infdis/jiu040 24443546 PMC4017367

[B233] Zetola N. M. Shin S. S. Tumedi K. A. Moeti K. Ncube R. Nicol M. (2014b). Mixed *Mycobacterium tuberculosis* complex infections and false- negative results for rifampin resistance by GeneXpert MTB/RIF are associated with poor clinical outcomes. *J. Clin. Microbiol.* 52 2422–2429. 10.1128/JCM.02489-2413 24789181 PMC4097703

[B234] Zhang G. Wang J. Yang J. Li W. Deng Y. Li J. (2015). Comparison and evaluation of two exome capture kits and sequencing platforms for variant calling. *BMC Genomics* 16:581. 10.1186/s12864-015-1796-1796 26242175 PMC4524363

[B235] Zhang S. Chen J. Cui P. Shi W. Shi X. Niu H. (2016). Mycobacterium tuberculosis mutations associated with reduced susceptibility to Linezolid. *Antimicrob. Agents Chemother.* 60 2542–2544. 10.1128/AAC.02941-2915 26810645 PMC4808145

[B236] Zhang S. Chen J. Cui P. Shi W. Zhang W. Zhang Y. (2015). Identification of novel mutations associated with clofazimine resistance in *Mycobacterium tuberculosis*. *J. Antimicrob Chemother* 70 2507–2510. 10.1093/jac/dkv150 26045528 PMC4539095

[B237] Zhang Z. Pang Y. Wang Y. Liu C. Zhao Y. (2014). Beijing genotype of *Mycobacterium tuberculosis* is significantly associated with linezolid resistance in multidrug-resistant and extensively drug-resistant tuberculosis in China. *Int. J. Antimicrob. Agents* 43 231–235. 10.1016/j.ijantimicag.2013.12.007 24439458

[B238] Zheng C. Li S. Luo Z. Pi R. Sun H. He Q. (2015). Mixed infections and rifampin heteroresistance among mycobacterium tuberculosis clinical isolates. *J. Clin. Microbiol.* 53 2138–2147. 10.1128/JCM.03507-3514 25903578 PMC4473183

[B239] Zignol M. Cabibbe A. M. Dean A. S. Glaziou P. Alikhanova N. Ama C. (2018). Genetic sequencing for surveillance of drug resistance in tuberculosis in highly endemic countries: a multi-country population-based surveillance study. *Lancet Infect. Dis.* 18 675–683. 10.1016/S1473-3099(18)30073-30072 29574065 PMC5968368

[B240] Zimenkov D. Nosova E. Y. Kulagina E. Antonova O. V. Arslanbaeva L. R. Isakova A. I. (2017). Examination of bedaquiline- and linezolid-resistant *Mycobacterium tuberculosis* isolates from the Moscow region. *J. Antimicrob Chemother* 72 1901–1906. 10.1093/jac/dkx094 28387862

